# Comprehensive analysis of the *MLP* genes in *Paulownia fortunei* and functional characterization of *PfMLP25* in response to pathogen invasion

**DOI:** 10.48130/forres-0026-0008

**Published:** 2026-03-31

**Authors:** Bingbing Li, Xiaoqiao Zhai, Haibo Yang, Aizhong Liu, Guoqiang Fan

**Affiliations:** 1 Institute of Paulownia, Henan Agricultural University, Zhengzhou, Henan 450002, China; 2 College of Forestry, Henan Agricultural University, Zhengzhou, Henan 450002, China; 3 Henan Province Academy of Forestry, Zhengzhou, Henan 450008, China; 4 Key Laboratory for Forest Resource Conservation and Utilization in the Southwest Mountains of China (Ministry of Education), College of Forestry, Southwest Forestry University, Kunming, Yunnan 650224, China

**Keywords:** *Paulownia fortunei*, Major latex-like protein 25, PfMYB100, Protein interactions, Immune formation, Ubiquitination

## Abstract

Major latex-like proteins (MLPs) play crucial regulatory roles in mediating plant responses to both abiotic and biotic stressors. Although MLPs have been identified in diverse plants, the genome-wide characteristics of MLP-encoding genes and their roles in pathogen defense in Paulownia (*Paulownia fortunei*) remain largely unexplored. *P. fortunei* is a fast-growing perennial tree widely cultivated across multiple regions of Asia due to its substantial economic and ecological value. Here, we identified 49 *PfMLP* genes from the *P. fortunei* genome and systematically characterized their sequence architectures and phylogenetic relationships. Transcriptome analyses revealed 16 *PfMLPs* that are responsive to Witches' Broom (PaWB) phytoplasma infection. Notably, we focused on the functional characterization of *PfMLP25,* whose expression was induced by PaWB infection. Heterogenous overexpression of *PfMLP25* in transgenic poplars enhanced pathogen resistance, confirming its critical role in anti-pathogen defense. Additionally, we demonstrated that the *PfMYB100* transcription factor regulates the transcription of *PfMLP25* and identified its interaction with two key immune system-essential proteins, PfCDPKa and PfRODa. Furthermore, we validated the interaction between PfMLP25 and the PfPUBa protein, indicating a potential role for PfMLP25 in the ubiquitination pathway during PaWB phytoplasma invasion. Taken together, our findings suggest that PfMLP25 functions by mediating immune system activation and participating in ubiquitination processes during PaWB phytoplasma invasion. This study presents new insights into the role of PfMLP25 and its underlying molecular mechanisms in plant pathogen defense.

## Introduction

Plants are frequently exposed to a wide variety of microorganisms, including bacteria, fungi, oomycetes, and viruses, which can negatively affect growth, development, and reproduction, leading to substantial yield losses in agricultural and forestry. To combat pathogen invasion, plants have evolved sophisticated, network-based defense systems that deploy chemical and molecular signals^[1]^. These systems include a primary layer of immune responses known as pattern-triggered immunity (PTI), which triggers a range of defensive reactions, such as alterations in membrane ion fluxes, accumulation of reactive oxygen species (ROS), and nitric oxide, callose deposition, and upregulation of pathogen-related (PR) gene expression^[2−4]^. The ubiquitination of proteins also plays a key role in modulating pathogen invasion and regulating the host immunity system to combat invasion^[5,6]^. However, due to the diversity of pathogens and plant responses to biotic stress, elucidating the molecular mechanisms of specific diseases is critical for developing targeted disease prevention strategies. Several signaling pathways, including those dependent on jasmonic acid (JA), salicylic acid (SA), and lignin biosynthesis pathways, as well as various regulatory factors (e.g., *SNCl*, *WRKY*, *MKK*, *HIR*, and *BAK1*), are consistently involved in orchestrating host plant defense against pathogen invasion^[7,8]^. Overall, plants utilize a broad range of defense tactics to counteract pathogen infections^[9−13]^.

In addition to these immune responses, plants produce stress-related proteins to mitigate the effects of biotic and abiotic stresses. Major latex-like proteins (MLPs), members of the Bet v 1 protein family, are stress-responsive proteins known to interact with multiple target proteins, initiating stress-associated signaling cascades. These proteins are widely implicated in mediating plant responses to various stressors^[14−16]^. MLPs typically possess a structure with three α-helices and seven *β*-sheets, along with a conserved glycine-rich loop (Gly-rich loop, G1yxG1yG1yxGlyThr)^[17,18]^. Due to their internal hydrophobic cavity, which confers broad binding affinities for hydrophobic molecules, MLPs often participate in controlling plant stress responses at both the physiological and molecular levels^[15,16]^. For instance, knockdown of *PbrMLP* expression significantly increased the susceptibility of pear seedlings to *Colletotrichum fructicola* infection, suggesting that *PbrMLP* is essential for anthracnose resistance in pears^[19]^. Similarly, loss-of-function of *CsMLP1* impaired the tolerance of *Cucumis sativus* to downy mildew, caused by the pathogen *Phytophthora melonis*^[20]^. Exogenous inoculation with *Verticillium dahliae* upregulated the expression of *GhMLP28*, which, in turn, promoted the expression of pathogenesis-related genes, ultimately enhancing disease resistance in cotton^[21]^. Furthermore, NbMLP43, targeted by *Potato Virus Y* (PVY), undergoes ubiquitin-mediated degradation, reducing tobacco's defense capacity against the virus^[22]^. Overexpression of *NbMLP423* in tobacco enhanced resistance to brown spot disease caused by the fungal pathogen *Alternaria alternata*^[23]^. These findings demonstrate that *MLP* genes are transcriptionally activated upon pathogen invasion and play a pivotal role in regulating host-pathogen interactions by integrating signaling cascades to enhance pathogen resistance and shape innate immune responses. However, the specific functional roles of individual MLP members may vary across plant species^[24]^.

Paulownia (*Paulownia fortunei*) is a multipurpose agroforestry tree known for its rapid growth, high-quality timber production, and strong adaptability to marginal lands. It is also adorned with lush foliage and vibrant flowers, which not only boast considerable ornamental appeal, but also harbor significant medicinal value^[25,26]^. This tree is widely cultivated in agroforestry systems throughout numerous regions of Asia, particularly in northern China, due to its remarkable economic and ecological values^[27,28]^. Despite its advantages, *P. fortunei* is susceptible to pathogen attack, which can lead to developmental abnormalities or even plant death. Paulownia Witches' Broom (PaWB) disease, caused by phytoplasma infection, has catastrophic impacts on host plants, causing severe physiological and morphological alterations that impair growth and productivity in agricultural and forestry systems^[29]^. A prior study showed that *PfMLP28* is involved in a competing endogenous RNA (ceRNA) regulatory network, modulating defense responses of Paulownia plants to PaWB phytoplasma infection^[30]^. However, the role of *MLP* genes in the immune response to PaWB phytoplasma infection in Paulownia remains largely unexplored. In this study, we conducted a genome-wide identification of *PfMLP* genes in *P. fortunei* and characterized their structural features. Through comparative transcriptomic analyses. We identified *PfMLPs* responsive to PaWB phytoplasma invasion. Specifically, we explored the functional characterization of *PfMLP25* and explored its potential molecular mechanism mediated by ubiquitination in immune response and pathogen resistance. This study adds novel insights into the molecular mechanism underlying the role of MLPs in plant immune responses and pathogen resistance, highlighting the specific function of *PfMLP25* in mediating immune defense in Paulownia against phytoplasma invasion.

## Material and methods

### Identification of *PfMLP* genes in *P. fortunei*

The Hidden Markov Model (HMM) file for the MLP domain (accession: PF00407) was retrieved from the Protein family (Pfam) database, and an HMM search was conducted against the *P. fortunei* genome. Homologous protein sequences of *MLP* genes in Paulownia were examined and redundant genes removed. The candidate protein sequences were validated by aligning them with the CDD, SMART, and Pfam databases to ensure they contained the typical structural domains of MLP proteins. The identified *MLPs* were named with the prefix 'Pf' (representing *P. fortunei*) followed by sequentially Arabic numerals starting from 1. The basic properties of the *PfMLP* genes were investigated using the ExPASy online tool (https://web.expasy.org/protparam). Subcellular locations of PfMLPs was predicted via the WoLF PSORT online tool (www.genscript.com/wolf-psort.html).

### Gene structures, conserved motifs, and phylogenetic analyses

Gene structures of the *PfMLPs* were visualized utilizing the Gene Structure Display Server (GSDS, http://gsds.cbi.pku.edu.cn), and conserved motifs were identified via MEME Suite (http://meme-suite.org/tools/meme). Results were visualized with Tbtools software (version 2.119). The amino acid sequences of PfMLPs and AtMLPs (from *Arabidopsis thaliana*) were aligned utilizing ClustalW. The phylogenetic tree was constructed using the neighbor-joining method with 1,000 bootstrap replicates in MEGA 7.0 software.

### Chromosome localization and *cis*-acting elements (CREs) analyses

The chromosomal localization of *PfMLP* genes was mapped using TBtools online software (version 2.119). The regulatory *cis*-acting elements in the promoter regions of *PfMLP* genes were predicted by analyzing the approximately 2.0-kb upstream sequence of each gene from the *P. fortunei* genome utilizing PlantCare (https://bioinformatics.psb.ugent.be/webtools/plantcare/html).

### Gene expression analysis and real-time qPCR (RT-qPCR) validation

Transcriptome data for *P. fortunei* were obtained from our previous study (NCBI accession numbers SRR11787882–SRR11787971, www.ncbi.nlm.nih.gov). Transcriptome data for *P. fortunei* seedlings treated with SA were also previously published^[31]^. The relative expression levels of each *PfMLP* were normalized utilizing RPKM values. A heatmap of the logarithm of RPKM value was plotted utilizing TBtools software (version 2.119).

RT-qPCR was applied to evaluate gene expression levels using the 2 × RealStar Fast SYBR qPCR Mix (Low ROX) kit, and *P. fortunei* cDNA as a template, with *Pfactin* (Paulownia_LG5G000560) as the internal control. The 2^−ΔΔCᴛ^ method was used to quantify gene expression. Results were averaged from three biological replicates, and statistical significance was determined using one-way ANOVA in SPSS software (version: 25.0), with * *p* < 0.05 and ** *p* < 0.01 considered statistically significant. All PCR primers are listed in Supplementary Table S1.

### Subcellular localization and generation of transgenic *Populus trichocarpa* lines overexpressing *PfMLP25*

To determine the subcellular localization of PfMLP25, its coding sequence was integrated into the pSAK277 vector. *Agrobacterium tumefaciens* strains carrying *PfMLP25* or the empty vector were co-infiltrated with a strain carrying the nuclear envelope marker AtSUN1-RFP^[32]^ into *Nicotiana benthamiana* leaves using a 1-mL needleless syringe. Tobacco seedlings were grown in a greenhouse under a light regime of 16 h of light and 8 h of darkness, at 28 °C and suitable humidity. After approximately 72 h, the injected leaves were observed using a high-resolution laser confocal microscope (A1HD25, NIKON, Japan) with an excitation wavelength of 488 nm, emission capture between 500−550 nm, 2.0% laser power, and a pinhole size fixed at 1.0 airy unit. Images were captured under consistent settings, with no post-acquisition processing except for minor brightness and contrast adjustments.

Wild-type *P. trichocarpa* was cultured in 350 mL plastic flasks containing woody plant medium (WPM) for 30 d, with the phytotron conditions set at a temperature of 25 ± 2 °C, light intensity of 130 µmol/m^2^/s, and a photoperiod of 16 h of light followed by 8 h of darkness. To generate *PfMLP25*-overexpressing *P. trichocarpa* lines, stems from wild-type were inoculated with *Agrobacterium tumefaciens* GV3101 carrying the *PfMLP25* gene at an OD600 of 0.6. The transformation protocol was adapted from Song et al.^[33]^.

### Bacterial pathogen invasion and histochemical detection

The *Pseudomonas syringae* pv. *tomato DC3000* (*Pst* DC3000) strain, obtained from China General Microbiological Culture Collection Center (CMCC) was grown overnight in Luria-Bertani medium with 25 μg/mL rifampicin at 28 °C. Bacteria were cultured, centrifuged, and resuspended, and modulated to the suitable concentration in 10 mM MgCl_2_. Transgenic *P. trichocarpa* plantlets were acclimatized for five weeks in pots with soil in a greenhouse before bacterial infection. The bacterial suspension was delivered into transgenic poplar leaves utilizing a syringe (similar to the manipulation in tobacco leaves), and the leaves were harvested to measure bacterial growth. Six leaf disks (three replicates) were ground in 100 μL of sterile H_2_O, and serial dilutions were plated on TSA medium supplemented with appropriate antibiotics with 1% tryptone, 1% sucrose, 0.1% glutamic acid, and 1.5% agar. After incubation at 28 °C for 3 d, bacterial colony-forming units (CFUs) were enumerated.

To visualize H_2_O_2_ accumulation, leaves were stained with 3,3-diaminobenzidine (DAB) solution (1 mg/mL, pH 7.5) as described previously^[34]^. After staining, the leaves were decolorized in boiling 95% ethanol and observed under an Olympus BX-51 microscope. Callose deposition was detected by aniline blue staining, as described by Wang et al.^[35]^ with minor adjustments. Leaves were inoculated for 72 h, fixed in acetic acid–ethanol solution (3:1) for 3 h to remove chlorophyll. Samples were subsequently immersed sequentially in 70% and 50% ethanol for 3 h each, then immersed in distilled water overnight. Leaf tissues were treated with 10% NaOH for 1 h to render them translucent. Thereafter, leaves were stained with 0.01% aniline blue and incubated under dark conditions for 3 h, followed by observation under a fluorescence microscope (M165 FC, Leica, Germany). *Pst* DC3000-inoculated leaves were also collected from WT and *PfMLP25-*overexpressing transgenic lines for H_2_O_2_ and callose quantification using the Hydrogen Peroxide (H_2_O_2_) Assay Kit (Comin, Suzhou, H_2_O_2_-2-Y) and Callose Content Assay Kit (Comin, Suzhou, PZZ-1-Y), following the manufacturer's protocols. The assays were performed with two biological replicates, where each replicate represented an independent plant.

### SA content detection

Endogenous SA was extracted from *P. trichocarpa* leaves 72 h post-inoculation. The SA concentration was quantified using an ELISA kit (Meimian, Jiangsu, China), following the manufacturer's instructions. SA levels were determined by measuring the optical density (OD) at 450 nm using a TECAN Spark microplate reader (TECAN, Männedorf, Switzerland), with the assay performed in accordance with the kit protocol.

### Yeast one-hybrid (Y1H) assay and dual-luciferase (LUC) reporter assay

The promoter sequence of *PfMLP25* and the coding sequence (CDS) of *PfMYB39*, *PfMYB41*, *PfMYB57*, *PfMYB100*, and *PfMYB128* were amplified by PCR and ligated into the pHIS2 and pGADT7 vectors, respectively (Supplementary Table S1). Yeast strain Y187 was co-transfected with plasmid for Y1H experiments to test DNA-protein interactions. The interaction assays were performed using a PEG-lithium acetate protocol. Transformed yeast cells were cultured on SD-Trp/-Leu/-His and SD-Trp/-Leu/-His with 30 mM 3-AT.

For dual-LUC assays, the *PfMLP25* promoter sequence was subcloned into the pGreenII0800-LUC vector, and the CDS of *PfMYB39*, *PfMYB41*, *PfMYB57*, *PfMYB100*, and *PfMYB128* were fused into pSAK277-Flag (Supplementary Table S1). The recombinant *Agrobacterium* strain GV3101 (pSoup-p19) carrying effector plasmids and reporter plasmids was infiltrated into four-week-old *N. benthamiana* leaves using needleless syringe inoculation. The leaves were harvested after 48 h, and the Dual-Luciferase® Reporter Assay System (Promega, Madison, USA) was utilized to measure the activities of fluorescence and *Renilla* luciferase.

### Identiﬁcation of proteins interacting with PfMLP25

The *PfMLP25* coding sequence was ligated into the pGS21T vector (TransGen Biotech) to express recombinant proteins. The purified proteins were obtained as described by Yuan et al^[36]^. Purified glutathione-*S*-transferase (GST)-PfMLP25 was incubated with crude protein extracts from *P. fortunei* for 2 h. Proteins that did not bind to GST-PfMLP25 were washed away using a buffer consisting of 300 mM NaCl, 50 mM NaH_2_PO_4_, and 30 mM iminazole, following the protocol by Gu et al.^[37]^. PfMLP25-interacting proteins were then identified via mass spectrometry. KEGG enrichment analysis was conducted utilizing the clusterProfiler software (https://github.com/YuLab-SMU/clusterProfiler) to identify significantly enriched pathways associated with the proteins interacting with PfMLP25.

### Yeast two-hybrid (Y2H) and bimolecular fluorescence complementation (BiFC) assays

Y2H assays were used to evaluate protein-protein interaction, as previously described^[38]^. The CDS of *PfMLP25* (1−146 aa), and truncated versions *PfMLP25*(1−45 aa, 46−120 aa, 121−146 aa, and 1−120 aa), along with *PfPUBa*, *PfCDPKa*, and *PfRODa*, were amplified into pGADT7 and pGBKT7 vectors, respectively. The CDS of *PfMLP25*, truncated *PfMLP25*(1−45 aa), *PfPUBa*, *PfCDPKa*, and *PfRODa* were further subcloned into pNC-ECN and pNC-ENN plasmids. The recombinant plasmids were introduced into *A. tumefaciens* and subsequently delivered into *N. benthamiana* leaves using syringe injection. The injected leaves were collected after 48–72 h for high-resolution laser confocal microscope monitoring. Nuclei were stained with 1% 4',6-diamidino-2-phenylindole (DAPI) solution (Solarbio, Beijing, China) for 7 min. The specific primers used in the Y2H and BiFC assays are listed in Supplementary Table S1.

### Cell-free degradation experiment

Total proteins were extracted from the apical bud of *P. fortunei* as previously described, with minor alterations^[37]^. GST-PfMLP25 was purified from *Escherichia coli* and separated into three adequate portions. These aliquots were incubated with the total protein extract at 25 °C. Two aliquots were treated with 40 μM of the 26S proteasome inhibitor MG132 (Solarbio, Beijing, China) and epoxomicin (GLPBIO, Montclai, USA), while the remaining aliquots served as a control. Samples were gathered at diverse time intervals and analyzed by Western blot, utilizing an anti-GST tag antibody (ProbeGene, Xuzhou, China) to quantify the abundance of PfMLP25.

### Ubiquitination assays *in vitro*

To produce His-PfMLP25 and GST-PfPUBa, the CDS of PfMLP25 and PfPUBa were inserted into the pGS21T and pGEX4T1 vector, respectively. The constructs were transferred into *E. coli* Rosetta (DE3) to generate recombinant proteins. Expression was induced with 0.5mM IPTG for 12 h at 16 °C. Ubiquitination assays were carried out using the PROTAC *in vitro* Ubiquitination Assay Kit (LifeSensors, PA, USA) protocol. PfMLP25 was detected via Western blot utilizing an anti-His antibody (ProbeGene PGM3004).

### Molecular docking

The homologous three-dimensional (3D) structures of the target proteins were predicted utilizing AlphaFold2^[39]^. For protein modeling and docking, Alphafold3 was utilized^[40]^. Molecular docking analyses were carried out to predict the conformations of the protein binding sites and to assess the interaction affinity. The AutoDockTools software was employed to conduct molecular docking analysis involving the receptor protein and two phytoplasma inhibitors, namely methyl methane sulfonate (MMS), and rifampicin (Rif).

## Results

### Identification, structural characterization, and phylogenetic implication of *PfMLP* genes in *P. fortunei*

**Figure 1:**
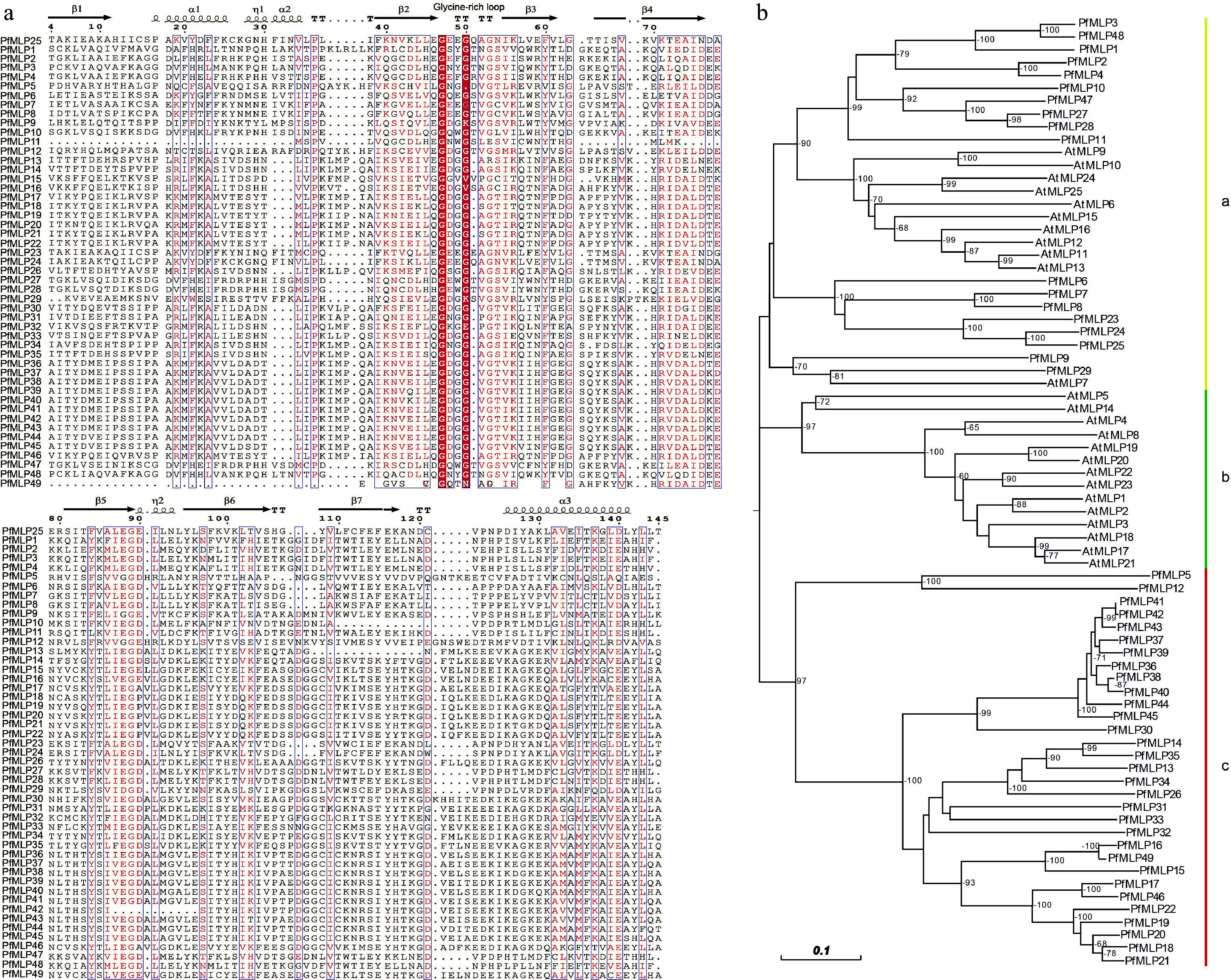
Sequence features of PfMLPs in Paulownia and phylogenetic relationships with Arabidopsis. (a) The multiple sequence alignment of PfMLPs with typical Bet v1 domains and conserved sequence structures. (b) Phylogenetic relationships of MLP members between *P. fortunei* and *A. thaliana*. Bootstraps (> 60%) were indicated.

Using the *P. fortunei* genome dataset, we identified a total of 49 *PfMLP* genes, each containing a complete Bet v1 allergen domain, which were designated as *PfMLP1* to *PfMLP49* based on their positions on chromosomes and scaffolds (Supplementary Fig. S1a). These *PfMLPs* ranged in length from 106 aa to 208 aa, with molecular weight (MW) spanning from 12.07 to 22.68 kDa. Detailed information pertaining to the identified PfMLP proteins is provided in Table 1. Upon examining the amino acid sequence and structural features of the PfMLP proteins, we found that all members share the typical structure of three *α*-helices, two *ȵ*s (*ȵ*1 and *ȵ*2), and seven *β*-sheets. Notably, a conserved glycine-rich loop with the consensus sequence GXXXXXG was consistently located between the *β*2 and *β*3 sheets, despite low sequence similarity among different PfMLP members (Fig. 1a). These conserved secondary structural features highlight structural conservation of PfMLPs, aligning with the canonical MLP structures reported in other plant species^[41]^. Based on amino acids sequences, we investigated the phylogenetic relationships between PfMLPs and AtMLPs (obtained from *Arabidopsis*), and constructed a neighbor-joining phylogenetic tree with three well-supported clades: a, b, and c (Fig. 1b). Interestingly, clade a comprised both AtMLPs and PfMLPs, whereas clades b and c were uniquely composed of AtMLPs and PfMLPs, respectively. Within clade a, the subclades were either AtMLPs-specific or PfMLPs-specific. These findings indicate a clear phylogenetic divergence between AtMLPs and PfMLPs. In terms of chromosomal distribution, the majority of *PfMLP* genes were clustered on specific chromosomes, particularly chromosomes 11 and 19 (Supplementary Fig. S1a). These observations indicate that most MLP proteins lack phylogenetic orthology between Arabidopsis and Paulownia evolutionary lineages.

**Table 1:** PfMLPs identified in the *P. fortunei* genome.

Gene	Gene ID	Amino acids	Molecular weight (KDa)	pI	Gravy	Subcellular localization
*PfMLP1*	*Pfo01g004220.1*	156	17.90	6.30	−0.096	chlo
*PfMLP2*	*Pfo03g006750.1*	152	17.53	5.30	−0.373	cyto
*PfMLP3*	*Pfo03g006760.1*	151	17.00	5.94	−0.231	nucl
*PfMLP4*	*Pfo03g006780.1*	162	18.55	5.21	−0.314	cyto
*PfMLP5*	*Pfo03g015030.1*	208	22.68	8.71	−0.212	chlo
*PfMLP6*	*Pfo06g009240.1*	147	16.08	4.53	0.232	cyto
*PfMLP7*	*Pfo06g009250.1*	148	16.15	4.83	0.538	cyto
*PfMLP8*	*Pfo06g009260.1*	148	16.10	5.14	0.555	cyto
*PfMLP9*	*Pfo07g005940.1*	150	16.81	5.20	−0.058	cysk
*PfMLP10*	*Pfo07g005960.1*	164	18.26	5.31	−0.039	cyto
*PfMLP11*	*Pfo07g006210.1*	106	12.07	5.37	0.066	cyto
*PfMLP12*	*Pfo08g013310.1*	205	22.88	5.05	−0.035	chlo
*PfMLP13*	*Pfo09g002850.1*	146	16.58	5.46	−0.364	cyto
*PfMLP14*	*Pfo11g002980.1*	160	17.76	5.00	−0.204	cyto
*PfMLP15*	*Pfo11g004160.1*	159	17.47	5.83	−0.219	cyto
*PfMLP16*	*Pfo11g004210.1*	160	17.93	5.65	−0.346	cyto
*PfMLP17*	*Pfo11g004220.1*	160	17.73	5.27	−0.221	nucl
*PfMLP18*	*Pfo11g004240.1*	160	17.93	5.09	−0.400	cyto
*PfMLP19*	*Pfo11g004250.1*	160	18.04	4.98	−0.422	cyto_nucl
*PfMLP20*	*Pfo11g004260.1*	160	17.92	5.11	−0.407	cyto
*PfMLP21*	*Pfo11g004270.1*	160	17.90	5.26	−0.457	nucl
*PfMLP22*	*Pfo11g004280.1*	160	17.95	4.96	−0.307	cyto
*PfMLP23*	*Pfo13g006930.1*	146	16.20	4.76	0.028	cyto
*PfMLP24*	*Pfo13g006940.1*	188	21.10	8.95	0.160	chlo
*PfMLP25*	*Pfo13g006950.1*	146	16.30	6.42	0.247	cyto
*PfMLP26*	*Pfo16g013080.1*	160	17.68	4.95	−0.206	cysk
*PfMLP27*	*Pfo17g000360.1*	155	17.70	4.74	−0.559	cyto
*PfMLP28*	*Pfo17g000370.1*	155	17.88	5.46	−0.598	cysk
*PfMLP29*	*Pfo17g005500.1*	152	16.86	4.90	−0.347	extr
*PfMLP30*	*Pfo19g001350.1*	161	17.62	6.13	−0.297	cyto
*PfMLP31*	*Pfo19g001360.1*	160	17.43	5.18	−0.378	cyto
*PfMLP32*	*Pfo19g001370.1*	160	18.37	5.69	−0.342	cyto
*PfMLP33*	*Pfo19g001380.1*	160	17.71	5.25	−0.252	nucl
*PfMLP34*	*Pfo19g004540.1*	159	17.67	4.96	−0.240	cyto
*PfMLP35*	*Pfo19g004550.1*	160	17.69	4.86	−0.303	chlo
*PfMLP36*	*Pfo19g006510.1*	156	17.02	5.49	−0.097	cyto
*PfMLP37*	*Pfo19g006520.1*	158	17.20	5.52	−0.130	cyto
*PfMLP38*	*Pfo19g006530.1*	158	17.19	5.49	−0.135	cyto
*PfMLP39*	*Pfo19g006540.1*	158	17.21	5.51	−0.099	cyto
*PfMLP40*	*Pfo19g006550.1*	158	17.16	5.66	−0.151	cyto
*PfMLP41*	*Pfo19g006560.1*	158	17.23	5.53	−0.090	cyto
*PfMLP42*	*Pfo19g006570.1*	147	16.12	6.20	−0.154	cyto
*PfMLP43*	*Pfo19g006600.1*	158	17.17	5.21	−0.083	cyto
*PfMLP44*	*Pfo19g006610.1*	158	17.23	5.65	−0.136	cyto
*PfMLP45*	*Pfo19g006620.1*	158	17.02	5.20	−0.130	cyto
*PfMLP46*	*Pfo19g006800.1*	160	17.77	5.00	−0.231	cyto
*PfMLP47*	*Pfoxxg001330.1*	153	17.51	5.28	−0.454	cyto
*PfMLP48*	*Pfoxxg035180.1*	151	17.01	5.57	−0.197	cyto
*PfMLP49*	*Pfoxxg039320.1*	117	13.00	4.61	−0.379	cyto

Analysis of the exon-intron organization revealed that most *PfMLP* genes carry one or two introns, with the rest being intronless. This suggests shared or analogous evolutionary trajectories among these genes (Supplementary Fig. S1b). Further analysis of conserved motifs identified 10 major motifs present across all PfMLP proteins (Supplementary Fig. S1c). Notably, we observed distinct differentiation in the distribution of these motifs across *PfMLP* genes in clade a and c: motifs 7, 4, 10, 5, and 6 were specifically associated with clade a, whereas motifs 2, 8, 1, 9, and 3 were exclusive to clade c. These observations suggest that the functions of genes in clade a and clade c may differ, though the specific roles of each motif remain uncertain. Subcellular localization predictions indicated that most of *PfMLPs* localize to the cytoplasm, with a subset targeted to the nucleus and chloroplasts (Table 1). These differential localization patterns of *PfMLPs* likely reflect their functional diversity in diverse subcellular organelles.

### Expression profiles of *PfMLPs* in mediating immune response to PaWB phytoplasma infection

**Figure 2:**
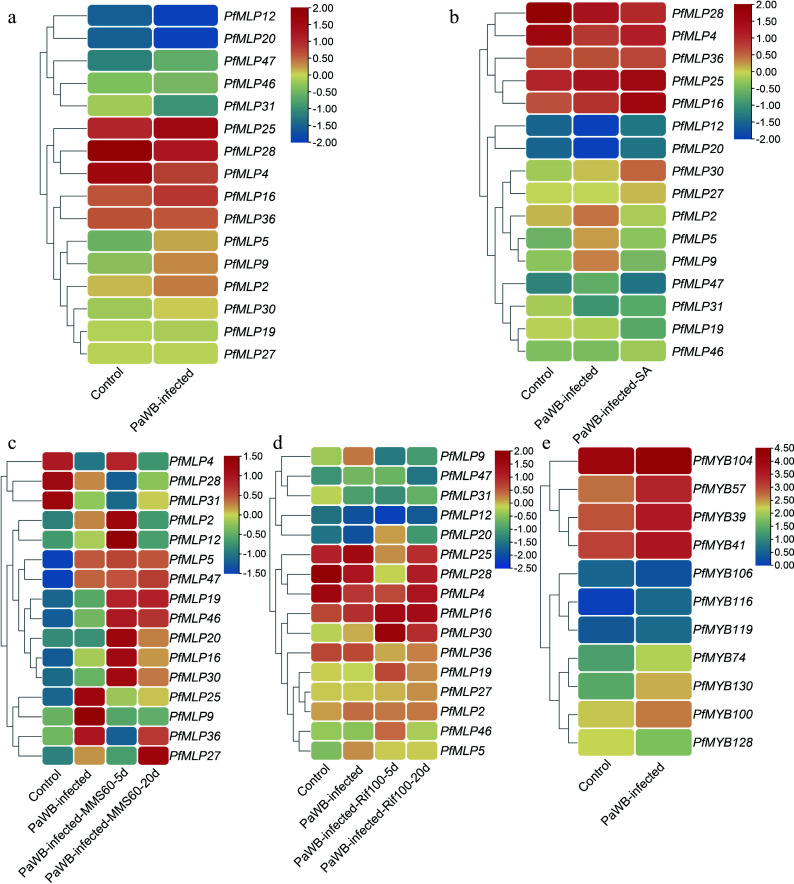
Expression profiles of *PfMLPs* under phytoplasma infection and different treatments of PaWB phytoplasma inhibitors. (a) Differential expression patterns of *PfMLPs* in response to PaWB phytoplasma invasion. (b) Expression patterns of *PfMLPs* in response to PaWB phytoplasma infection under exogenous SA treatment. (c) Expression patterns of *PfMLPs* in response to PaWB phytoplasma infection under methyl methanesulfonate (MMS) treatment in PaWB-infected seedlings. Expression changes of *PfMLPs* were examined at 5 and 20 d post-treatment, respectively. (d) Expression patterns of *PfMLPs* in answering to PaWB phytoplasma infection under rifampicin (Rif) treatment in PaWB-infected seedlings. Expressional dynamics of *PfMLPs* were examined at 5 and 20 d post-treatment, respectively. (e) Differential expression patterns of *PfMYB*s under phytoplasma stress. Relative expression values were normalized, with the transcriptomics data range colored from low (blue) to high (red).

To investigate which *PfMLP* genes contribute to defense against PaWB phytoplasma infection, we performed comparative transcriptome analyses of apical bud tissues from both PaWB-infected and healthy Paulownia seedlings. Our comparative transcriptome analyses revealed that 16 *PfMLP* were differentially expressed genes (DEGs) (*p* < 0.05, |log2FoldChange| > 1) in PaWB-infected seedlings: nine were transcriptionally upregulated, including *PfMLP 2, 5, 9, 16, 25, 30, 36, 46,* and *47,* and seven were transcriptionally downregulated, including *PfMLP 4, 12, 19, 20, 27, 28,* and *31* (Fig. 2a). These results suggest that these *PfMLPs* are specifically involved in the defense against PaWB phytoplasma infection. Given that PaWB-infected seedlings can be rescued through treatment with SA and the phytoplasma inhibitors MMS and Rif^[28,31]^, we further investigated the transcript level changes of these 16 *PfMLPs* in response to these treatments during disease rescue. As reported previously, exogenous SA application to the apical buds of PaWB-infected seedlings for 30 d, or treatment with 60 mg/L MMS or 100 mg/L Rif for 5 and 20 d, resulted in a distinct alleviation, and in some cases compete disappearance of Witches' Broom symptoms in the infected plants^[28,31]^. We utilized transcriptome data generated in our previous study^[31]^ to compare the expression changes of the 16 *PfMLPs* in response to SA-mediated disease rescue. Our results showed that *PfMLP16*, *PfMLP25*, *PfMLP30*, and *PfMLP46* were significantly upregulated, whereas *PfMLP2*, *PfMLP5*, *PfMLP9*, and *PfMLP19* were downregulated during SA-mediated disease rescue (Fig. 2b). Next, we utilized transcriptome data from Cao et al.^[28]^ to compare the expression changes of these 16 *PfMLPs* during PaWB disease rescue following treatment with 60 mg/L MMS. This analysis revealed transcriptional alterations in most of the 16 *PfMLPs*, including the upregulation of *PfMLP19*, *PfMLP25*, and *PfMLP46*, the downregulation of *PfMLP9*, and variable expression of *PfMLP16*, *PfMLP20*, *PfMLP27*, *PfMLP30*, and *PfMLP36* (Fig. 2c). Similarly, we analyzed the expression changes of the 16 *PfMLPs* during PaWB disease rescue with 100 mg/L Rif treatment. We found that *PfMLP4*, *PfMLP16,* and *PfMLP30* were upregulated, while *PfMLP5*, *PfMLP9*, and *PfMLP36* were downregulated, and *PfMLP25*, *PfMLP28*, and *PfMLP46* showed variable changes during Rif-mediated rescue (Fig. 2d)^[28]^. To validate these transcriptomic findings, we performed RT-qPCR on selected *PfMLP* genes that exhibited transcriptional alterations in response to PaWB infection and following treatment with SA, MMS, and Rif treatments. The RT-qPCR results confirmed the transcriptomic data, demonstrating consistent changes in gene expression during PaWB infection and rescue treatments (Supplementary Fig. S2). These results indicate that *PfMLPs* exhibit diverse expression patterns responding to PaWB phytoplasma infection and subsequent disease rescues, implying their involvement in diverse transcriptional and regulatory pathways throughout the infection and rescues processes.

To investigate the CREs in the promoter regions of the 16 *PfMLP* genes, we retrieved the −2,000 bp upstream DNA sequences before the transcription start sites from the *P. fortunei* genome. We then predicted potential CREs and identified a diverse set of elements, including multiple hormone responsiveness elements (for abscisic acid, gibberellin, SA, and auxin), defense and stress responsiveness elements, as well as MYB binding sites (Supplementary Fig. S3). These findings suggest that *PfMLPs* may be regulated by diverse factors during the response to PaWB phytoplasma infection and disease rescues.

### Functional analyses of *PfMLP25* in response to pathogen infection

**Figure 3:**
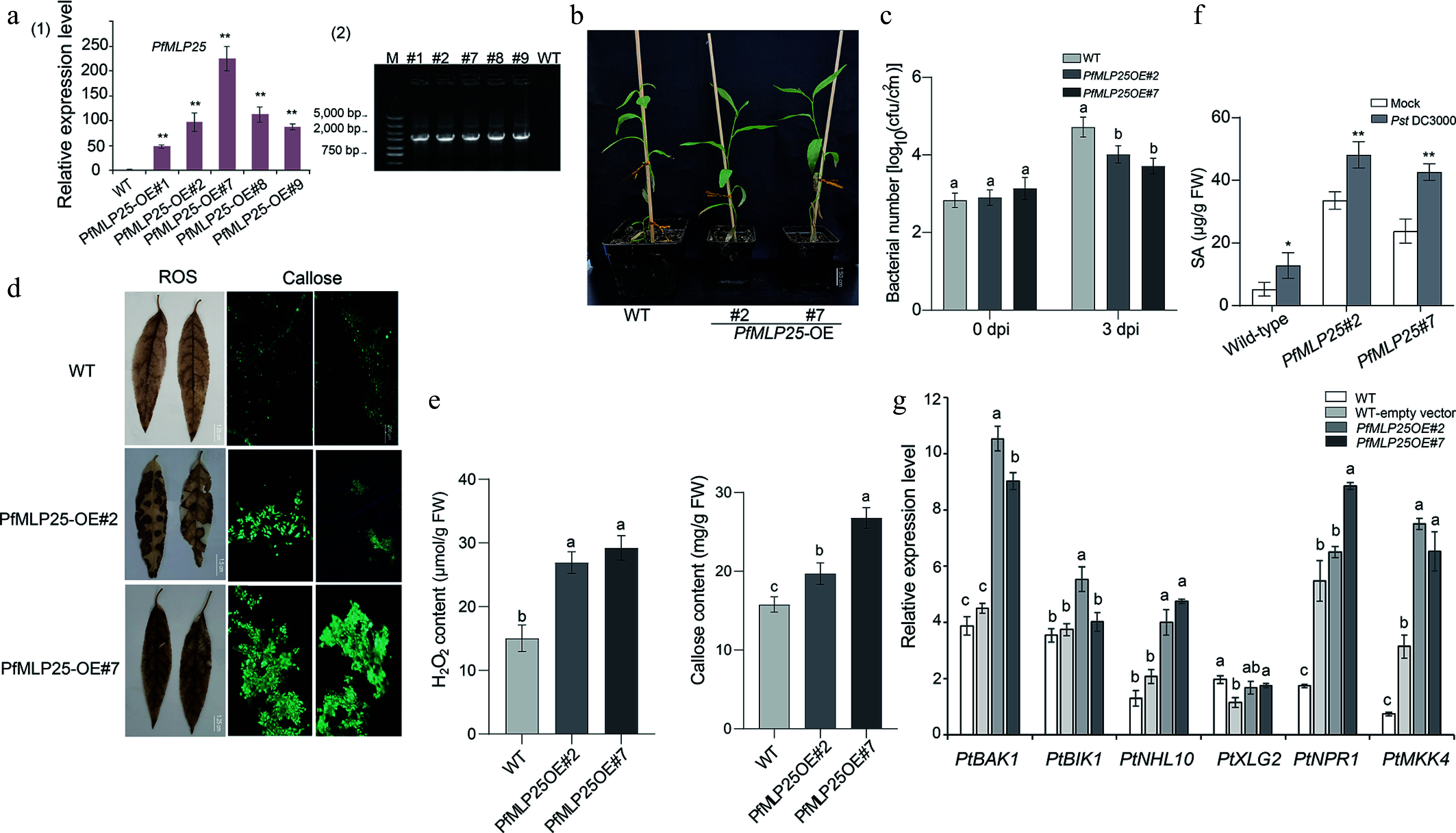
Heterologous overexpression of *PfMLP25* in poplar enhanced pathogen resistance in the transgenic lines. (a) Transcriptional changes in *PfMLP25* across different PfMLP25-overexpressing poplar lines (#1, #2, #7, #8, and #9) compared to the wild type (WT), with data generated from three technical replicates and statistical significance determined (* *p* < 0.05, ** *p* < 0.01) (1) and PCR validated positive transformants (2). (b) Phenotypes of transformants #2 and #7 did not exhibit visible difference with WT. (c) Growth rates of *Pst* DC3000 bacterial in infected leaves of transformants and WT. Leaves of 5-week-old poplars seedlings from transformants #2, #7 and WT were infiltrated with suspensions of *Pst* DC3000, and bacterial numbers were assessed at 0 and 3 dpi (day post inoculation). (d) Enhancement of H_2_O_2_ accumulation and callose deposition in transformants poplar leaves. Transformants' leaves (OE#2 and OE#7) were treated with 3,3'-diaminobenzidine after they were infiltrated with suspensions of *Pst* DC3000 for 72 h. Two transformants (OE#2, OE#7) exhibited higher contents of H_2_O_2_ indicated by brown insoluble polymer and more callose deposition indicated by bright-blue spots, which were generated from treatments of the stain aniline blue and imaged under bright-field UV, Bar = 200 μm. (e) Comparisons of quantified H_2_O_2_ and callose contents between transformants and WT. (f) The endogenous SA content in WT and over-expressed transformants plants inoculated with *Pst* DC3000 or mock-treated (water). Bar chart represents mean ± SE (n = 3 biological replicates of six plants for each plant line and treatment), with statistical significance (* *p* < 0.05, ** *p* < 0.01). (g) Expressional comparisons of defense-related marker genes between transformants and WT using a qRT-PCR technique. Data presented were means of triple repeats with standard deviation (n = 3). Each biological replicate consisted of pooled samples from three leaves. The normality of the data was assessed using a Q-Q plot. The letters denote statistical significance differences as determined by one-way ANOVA (*p* < 0.05).

Given the differential expression of *PfMLP25* in response to PaWB phytoplasma infection and subsequent disease rescues, we focused on its functional characterization. First, we investigated the subcellular localization of *PfMLP25* and found that it localized to the cytoplasm (Supplementary Fig. S4). Due to the unavailable transformation system in Paulownia, we heterogenously overexpressed *PfMLP25* in poplar (*P. trichocarpa*) to test its function during pathogen invasion. Five PfMLP-overexpressing transformants (OE#1, OE#2, OE#7, OE#8, OE#9) were successfully obtained and validated via PCR and qRT-PCR (Fig. 3a). Phenotype analysis revealed no visible differences between the overexpressing lines and wild-type (WT) plants, as exemplified by the randomly selected lines OE#2 and OE#7 (Fig. 3b). To evaluate the defense response, we inoculated leaves from *PfMLP25*-overexpressing transformants and WT plants with *Pst* DC3000 for 72 h. The results showed that the overexpressing lines exhibited enhanced resistance to *Pst* DC3000, with a significant reduction in bacterial growth relative to WT (Fig. 3c; *p* < 0.05). Since ROS burst and callose deposition are key markers for assessing enhanced defense capacity in transgenic lines, we quantified ROS levels and callose accumulation in the inoculated leaf tissues of *PfMLP25*-overexpressing transformants, and WT controls. Results showed that ROS and callose levels were significantly higher in the transformant tissues compared to the controls (Fig. 3d, e; *p* < 0.05). When quantifying SA concentrations in *Pst* DC3000-inoculated WT and *PfMLP25*-overexpressing transformants, we found that SA levels were significantly higher in the inoculated lines than in mock-treated controls at 72 h post-inoculation. Notably, *PfMLP25*-overexpressing transformants accumulated significantly more SA than the WT (Fig. 3f). These results, combined with the reduced bacterial growth, elevated ROS burst, and increased callose deposition, indicate that *PfMLP25-*overexpressing transformants exhibit enhanced defense capacity in response to pathogen infection. Host immune response genes such as *PtXLG2*, *PtBAK1*, *PtNHL10*, *PtMKK4*, *PtNPR1*, and *PtBIK1* are typically activated in response to pathogen infection in *P. trichocarpa*^[42]^. To investigate whether *PfMLP25* plays a role in immune system activation in transgenic lines, we quantified the transcriptional changes of these genes in *PfMLP25-*overexpressing transformants and WT plants via qRT-PCR. As shown in Fig. 3g (*p* < 0.05), most of these immune response genes, including *PtBAK1*, *PtNHL10*, *PtMKK4*, and *PtBIK1*, were significantly upregulated in the *PfMLP25-*overexpressing lines (OE#2 and OE#7), suggesting that *PfMLP25* participates in the activation of the immune system in *P. trichocarpa* during pathogen infection.

### PfMLP25 transcription is regulated by PfMYB100

**Figure 4:**
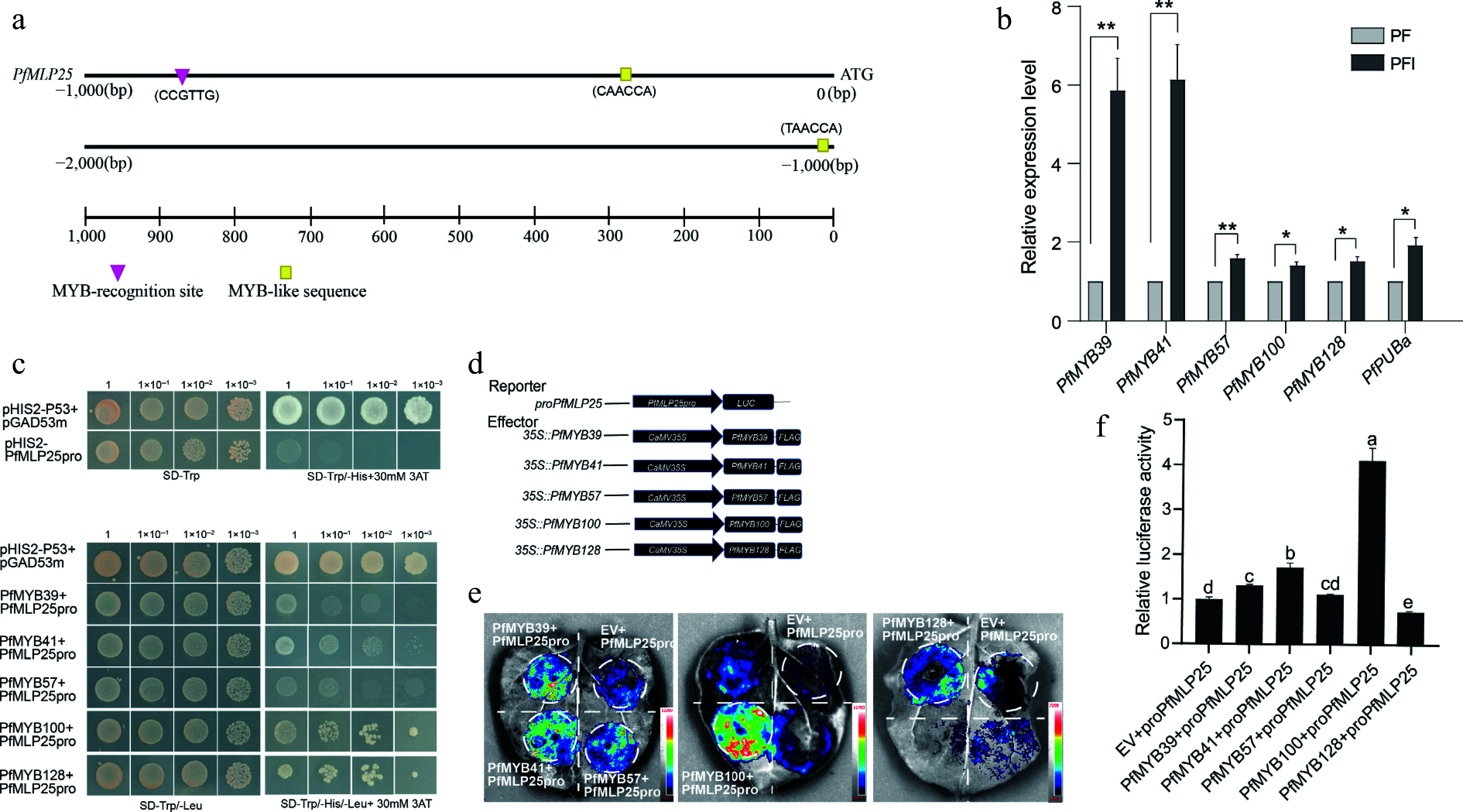
The expression of *PfMLP25* is regulated by transcript factor PfMYB100. (a) The 2,000 bp upstream promoter region of PfMLP25 harbored MYB recognition and binding sites. (b) *PfMYBs* genes are upregulated after PaWB phytoplasma infection based on the qRT-PCR technique. Data presented were means with standard deviation (*n* = 3) (* *p* < 0.05; ** *p* < 0.01). Statistical analysis was performed using an independent samples t-test. (c) Yeast one-hybrid assay utilizing pGADT7-PfMYBs as the prey and pHIS-PfMLP25 as the bait. Following co-transformation with recombinant vectors, yeast cells were plated on SD/-Trp/-Leu selection medium and subjected to serial dilutions (10-fold gradients). Five μL aliquots of each dilution were spotted onto SD/-Trp/-Leu/-His plates supplemented with 30 mM 3-AT. After 72 h incubation at 30 °C, colony growth was visually inspected. (d) Schematic diagram of vectors for the dual-luciferase reporter assay. (e) A representative LUC signal image in tobacco leaves. (f) Quantified LUC signal presented relative LUC activity. Letters indicate statistically significant differences determined by one-way ANOVA (*p* < 0.05).

To investigate the potential molecular mechanism regulating *PfMLP25* expression in Paulownia, we first analyzed the *cis*-elements in the promoter region of *PfMLP25*, which spans a 2-kb upstream sequence before the translation initiation site. We found that the *PfMLP25* promoter sequence (PfMLP25pro) contains typical MYB-recognition sites and several MYB-like binding sites, including the core sequences 'CCGTTG', 'CAACCA', and 'TAACCA' (Fig. 4a). These findings strongly imply that *PfMLP25* may be regulated by MYBs. In the above comparative transcriptomic analyses, we identified several *PfMYB* transcription factors*,* including *PfMYB39*, *PfMYB100*, and *PfMYB116*, which were differentially expressed in response to PaWB phytoplasma infection (Fig. 2e). To validate which of these PfMYBs regulate *PfMLP25* transcription, we performed qRT-PCR and confirmed the differential expression of *PfMYB39* (Paulownia_LG5G001379)*, PfMYB41* (Paulownia_LG5G001535)*, PfMYB57* (Paulownia_LG6G000458)*, PfMYB100* (Paulownia_LG11G000996)*,* and *PfMYB128* (Paulownia_LG17G000049) during PaWB infection (Fig. 4b; *p* < 0.05). We then focused on investigating which of these *PfMYBs* directly regulate *PfMLP25* expression during PaWB infection. Y1H assays confirmed that *PfMYB39*, *PfMYB41*, *PfMYB57*, *PfMYB100*, and *PfMYB128* can directly bind to the promoter region of *PfMLP25,* suggesting they might regulate its expression (Fig. 4c). Following vector construction, we performed dual-Luciferase (dual LUC) assays (Fig. 4d). Co-transfection of *35S::PfMYB100* with *PfMLP25pro:*LUC resulted in a marked upregulation of luciferase activity, as evidenced by enhanced luminescence signals (Fig. 4e, f). These results confirm that *PfMYB100* directly binds to the *PfMLP25* promoter and activates its expression in tobacco leaf. Collectively, these findings clearly reveal that *PfMLP25* is transcriptionally regulated by *PfMYB100*.

### Potential mechanism underlying *PfMLP25*-mediated immune system activation

**Figure 5:**
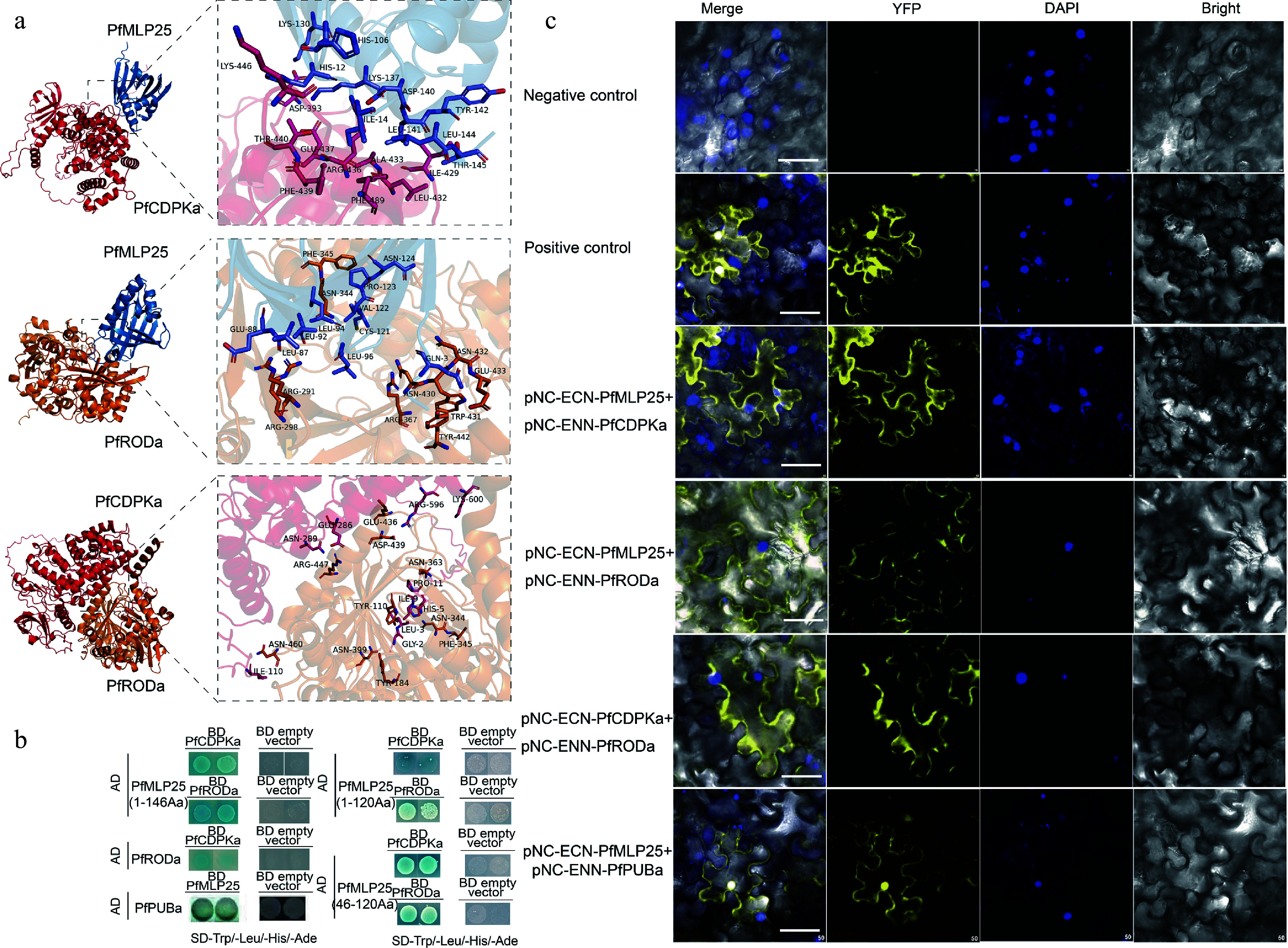
Verification of protein interactions between PfMLP25 and PfCDPKa/PfRODa. (a) Molecular docking analysis revealed pairwise interactions of PfMLP25 and PfCDPKa, PfMLP25 and PfRODa, and PfCDPKa and PfRODa. The docking simulations are performed using Alphafold3, and the binding affinities are presented as binding free energies (kcal/mol). (b) Yeast two-hybrid assays confirm the interactions between PfMLP25 and PfCDPKa, PfMLP25 and PfRODa, as well as PfCDPKa and PfRODa. Blue yeast colonies on SD/-Trp-His-Leu-Ade medium supplemented with 20 μg/mL X-*α*-Gal (-THLA+X-*α*-Gal) serves as an indicator of the protein-protein interactions. (c) Bimolecular fluorescence complementation visualization of the interactions between PfMLP25 and PfCDPKa, PfMLP25 and PfRODa, PfCDPKa and PfRODa, as well as PfMLP25 and PfPUBa. Cell nuclei were stained by DAPI (in blue). pNC-ENN-PfARF13 and pNC-ECN-PfAux/IAA45 were used as a positive control. Truncated non-functional PfMLP25 proteins were used as a negative control. Bar scale, 50 μM.

Since MLPs commonly interact with numerous target proteins to initiate metabolism and signaling pathways, identifying the protein interactors of PfMLP25 is essential for elucidating the potential molecular mechanism underlying its functions in response to PaWB infection. We used recombinant PfMLP25 as bait to perform a GST pull-down coupled with MS analysis to identify its interacting proteins from PaWB-infected seedlings. This approach identified 156 PfMLP25-interacting proteins (Supplementary Fig. S5; Supplementary Table S2). To further inspect the potential roles of these identified proteins, we performed KEGG (Kyoto Encyclopedia of Genes and Genomes) pathway enrichment analysis, showing that these proteins were significantly enriched in several key biological pathways such as defense response pathways and hormone signal transduction (Supplementary Fig. S6a). Specifically, these proteins functionally participate in diverse biological processes such as defense and stress responses, photosynthesis, transcription and translation, hormone signal transduction pathways, primary metabolism and protein modification (Supplementary Fig. S6b). These findings suggest that PfMLP25 participates in manifold biological processes and metabolic pathways. Notably, we identified two key enzymes—PfCDPKa, a CDPK-related kinase 1-like protein (Paulownia_LG15G000051.1), and PfRODa, a reticuline oxidase-like protein (Paulownia_LG8G000155.1)—which play crucial roles in immune system activation during plant defense and stress responses^[43,44]^. To further explore the interactions between PfMLP25 and these two proteins, we conducted molecular docking analyses. Results revealed that both PfCDPKa and PfRODa docked efficiently with the conserved domain of PfMLP25, forming stable hydrogen bonds and exhibiting strong binding affinities, with binding energies of −34.39 kcal/mol and −41.87 kcal/mol, respectively (Fig. 5a). Additionally, PfCDPKa also docked effectively with PfRODa, with a binding energy as low as −44.89 kcal/mol (Fig. 5a). To pinpoint the specific regions of PfMLP25 involved in protein-protein interactions, we truncated the full-length PfMLP25 protein into five segments: 1−45 aa, 46−120 aa, 121−146 aa, 1−120 aa, and 1−146 aa. Y2H experiments demonstrated that the 46−120 aa, 1−120 aa, and 1−146 aa fragments interacted with both PfCDPKa and PfRODa, suggesting that this 46-120 aa region is critical for interacting with these two proteins (Fig. 5b). Similarly, the BiFC experiment also confirmed these findings, as visible fluorescent signals were detected as the cell membrane in tobacco leaf cells co-expressing PfMLP25 + PfCDPKa, PfMLP25 + PfRODa, and PfRODa + PfCDPKa (Fig. 5c). These results clearly indicate that PfMLP25 interacts with both PfCDPKa and PfRODa, revealing its involvement in immune system activation during PaWB phytoplasma invasion.

**Figure 6:**
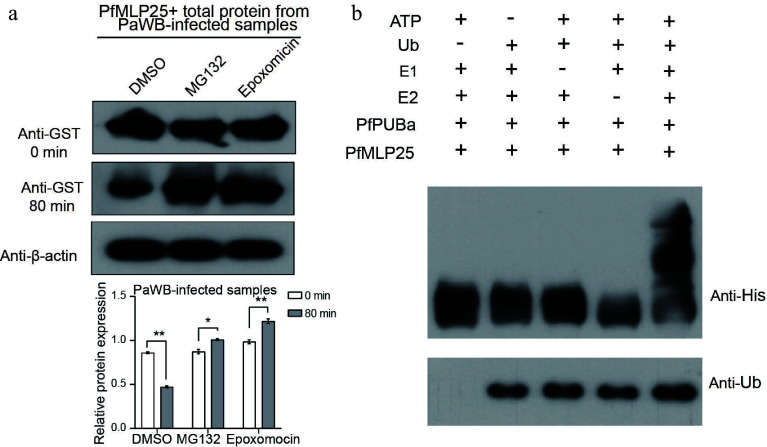
PfPUBa interacts with PfMLP25 and functions as an E3 ubiquitin ligase to modulate the ubiquitinate of PfMLP25 *in* *vitro*. (a) Cell-free degradation assay of recombinant GST-PfMLP25 protein. Recombinant GST-PfMLP25 protein was purified from *E. coli* and cultured with proteins from PaWB-infected Paulownia, and treated with specific 26S proteasome inhibitors MG132, and epoxomicin at different time intervals. Western blot analysis was performed using an anti-GST antibody, with anti-β-actin utilized as a control. The bands were examined using ImageJ software, and the results were presented as mean with standard deviation (*n* = 3). Statistical significance is denoted by * *p* < 0.05 and ** *p* < 0.01. (b) PfPUBa functions as an E3 ligase of PfMLP25 *in vitro*. GST-PfPUBa and His-PfMLP25 fusion proteins were purified. The E3 activity of the GST-PfPUBa fusion protein was detected under conditions with or without the addition of ATP, ubiquitin, ubiquitin-activating enzyme (E1), ubiquitin-conjugating enzyme (E2), His-PfMLP25, or ubiquitin (Ub).

Protein ubiquitination is a widespread post-translational modification that plays a key role in regulating immune activation or suppression during pathogen invasion^[45,46]^. We noted that three proteins contained U-box domains, a conserved domain in ubiquitination-related proteins, implying that PfMLP25 likely functions in the ubiquitination pathway. According to sequence features and homology to *Arabidopsis*, we found that these three proteins (Paulownia_LG15G001218.1, Paulownia_LG10G000789.1 and Paulownia_LG8G001932.1) contained U-box or RING finger domains (Supplementary Table S2), key functional domains of E3 ubiquitin-protein ligases, and we designated them as PfPUBa (Paulownia_LG15G001218.1), PfPUBb (Paulownia_LG10G000789.1) and PfPUBc (Paulownia_LG8G001932.1). Among them, PfMLP25 specifically interacted with PfPUBa, as confirmed by Y2H and BiFC experiments (Fig. 5b), while it did not interact with PfPUBb or PfPUBc. Additionally, the transcript level of *PfPUBa* was significantly upregulated under PaWB stress conditions (Fig. 4b). To validate whether PfMLP25 abundance is regulated by the PfPUBa-mediated ubiquitination pathway, we conducted an *in vitro* cell-free degradation assay. We purified GST-tagged PfMLP25 protein from *E. coli* and incubated it with crude total protein extracts from PaWB-infected Paulownia seedling buds. The purified PfMLP25 protein was treated with DMSO (control), and a specific ubiquitin-proteasome inhibitor MG132, or epoxomicin, respectively. We found that PfMLP25 abundance was higher in the MG132 and epoxomicin treatment groups, compared to the DMSO control at 80 min post-treatment (Fig. 6a). Further, to investigate whether PfPUBa acts as an E3 ubiquitin ligase for PfMLP25 *in vitro,* cell-free ubiquitination assays were conducted by supplementing ATP, ubiquitin (Ub), ubiquitin-activating enzymes (E1), and ubiquitin-conjugating enzymes (E2) in the presence of PfPUBa^[47,48]^. Results demonstrate that PfPUBa acts as an E3 ubiquitin ligase to ubiquitinate PfMLP25 (Fig. 6b). Taken together, these results indicate that PfMLP25 is ubiquitinated by PfPUBa, which serves as an E3 ubiquitin ligase during PaWB phytoplasma invasion.

### Molecular docking of PfMLP25 with Rif and MMS

As demonstrated above, the interaction between PfPUBa and PfMLP25 promotes the degradation of PfMLP25, which likely attenuates the resistance and defense responses of Paulownia plants to PaWB. The PfMLP25 protein, which is associated with immune-related gene expression in Paulownia, was subjected to molecular docking simulations with MMS and Rif (Supplementary Fig. S7). PfMLP25 docked effectively with MMS, forming stable hydrogen bonds between MMS and the residues PHE-98, PHE-94, and ALA-86. Similarly, PfMLP25 docked effectively with Rif, exhibiting a significant binding affinity with a low potential energy of −6.5 kcal/mol. These *in silico* results confirm that Rif and MMS bind to PfMLP25.

## Discussion

As mentioned above, MLPs constitute a unique plant-specific gene family that is widely involved in adjusting plant growth and development, modulating diverse metabolism pathways, and responding to biotic and abiotic stresses^[21,49,50]^. Genome-wide identification of MLP-encoding genes has been performed in several plant species, such as *A.* *thaliana* (25 members), *Populus tremula* (10 members), *Rosa chinensis* (46 members), *Prunus persica* (30 members), and *Arachis hypogaea* (68 members)^[41,51,52]^. Here, we identified 49 MLP members in Paulownia. The varied number of *MLPs* could be related to genome size and different evolutionary histories in plants. Indeed, while the sequence architecture of MLPs is conserved, their primary sequence similarity is low, with high secondary structural conservation. This observation aligns with findings in *Cucumis sativus,* where similar patterns were observed^[20]^. Phylogenetic analysis of MLP proteins between Arabidopsis and Paulownia revealed distinct phylogenetic relationships among most family members, identifying separate Arabidopsis- and Paulownia-specific clades. Likewise, phylogenetic studies of MLPs between apple (*Malus domestica*) and *Arabidopsis*, as well as between roses (*Rosa chinensis*) and *Arabidopsis*, also exhibited that many MLP members were phylogenetically differentiated^[41,53]^. These results underscore that MLPs are phylogenetically and evolutionarily divergent, implying that their functions may be non-conserved across different species.

The primary objective of this study was to identify key *PfMLP* genes involved in mediating pathogen responses and activating immune system during PaWB phytoplasma invasion. In this regard, we identified that 16 *PfMLPs* manifested altered expression patterns, suggesting these genes function in the Paulownia response to PaWB phytoplasma invasion. Prior studies have reported that PaWB-infected seedlings can be rescued by exogenous treatment with MMS, Rif and SA. Accordingly, we observed divergent expressional changes in these 16 *PfMLPs* following exogenous MMS, Rif and SA application. These results imply that the 16 *PfMLPs* likely contribute to immune system activation in Paulownia and/or play a role in different pathways that underpin the rescue of PaWB-infected plants.

Based on its upregulated expression in response to PaWB phytoplasma invasion and differential expression patterns following exogenous MMS, Rif, and SA treatments, we focused on investigating the regulatory mechanisms and biological functions of *PfMLP25.* Interestingly, we found that *PfMLP25* is directly regulated by the transcription factor *PfMYB100*, though regulatory factors likely vary among individual *PfMLP* family members. Similarly, *NtMYB108* has been identified as a regulator of *NtMLP423* expression in tobacco under chilling stress^[54]^. Furthermore, *GhMYB108* and *AtMYB30* have been shown to be vital regulators in the immune response against pathogen attack^[55,56]^. These outcomes suggest that MYB transcription factors are likely key regulators of *MLP* gene expression in plants. Notably, heterologous overexpression of *PfMLP25* in poplar clearly demonstrated that this gene modulates physiological processes and enhances pathogen defense responses in the host. However, due to the lack of an established transformation system and virus-mediated transient expression techniques (e.g., gene silencing) in Paulownia, we validated the function of *PfMLP25* in heterologously transformed poplar instead. Although the current functional evidence for *PfMLP25* comes from heterologously transformed in poplars, these results, combined with its transcriptional dynamics in response to pathogen invasion provide strong support for its role in enhancing pathogen defense in Paulownia. Similarly, *NbMLP43* was found to confer resistance to *PVY* invasion in tobacco^[22]^. In *Brassica napus*, *BnMLP6* acts as a key defense gene that encodes a plasma membrane- and endoplasmic reticulum-localized protein. This protein interacts with NPF5.12 to enhance suberin deposition, thereby restricting the infection and systemic spread of *Verticillium longisporum*^[57]^**.** Once a genetic transformation system for Paulownia is established, the function of *PfMLP25* can be further confirmed in the native host.

Protein-protein interactions are key to the functional mechanism of individual proteins. In this study, we identified 156 PfMLP25-interacting proteins from PaWB-infected seedlings. Functionally, these proteins are implicated in manifold biological pathways, including defense and stress responses, photosynthesis, transcription and translation, hormone signaling, primary metabolism, and protein modification, suggesting that PfMLP25 exerts multifunctional roles in response to PaWB phytoplasma invasion. Host responses to pathogen invasion are inherently coupled to immune system activation, and we accordingly identified interactions between PfMLP25 and PfCDPKa/PfRODa, two key enzymes associated with plant immune system activation. Following experimental validation of the PfMLP25-PfCDPKa/PfRODa interactions, it is reasonable to infer that PfMLP25 contributes functionally to immune activation in Paulownia, although the underlying molecular mechanisms remain to be elucidated.

Given that protein ubiquitination is a widespread post-translational modification that mediates pathogen defense and immune system activation in response to pathogen invasion^[5,58,59]^, we emphasize the interaction between PfMLP25 and PfPUBa, an E3 ubiquitin ligase implicated in protein ubiquitination. Experimental evidence clearly indicates that PfMLP25 abundance is modulated by ubiquitination, with PfPUBa serving as the E3 ubiquitin ligase that regulates this process during PaWB phytoplasma invasion. Similarly, NbMLP43, a tobacco protein associated with enhanced pathogen defense responses, also exhibits reduced abundance via ubiquitination^[54]^. In a parallel example, the E3 ligase StPUB17 positively regulates immune responses by targeting and degrading StKH17, a negative immune regulator in *Solanum tuberosum*^[60]^. Additionally, *in silico* molecular docking analyses suggested that the conserved Bet v1 domain of PfMLP25 likely serves as a binding site for Rif and MMS. Predicted hydrogen bond formation at these sites supports the potential for ligand binding to alter the protein's properties. This also provides a testable hypothesis for future studies aimed at investigating the precise molecular mechanisms underlying the chemical rescue of PaWB-infected plants. However, the functional role of PfMLP25 ubiquitination in mediating pathogen defense and immune activation during PaWB phytoplasma invasion warrants further investigation.

## Conclusions

In conclusion, we identified 49 *PfMLP* genes from the *P. fortunei* genome and characterized their sequence architecture and phylogenetic relationships. Comparative transcriptomic analyses revealed that 16 *PfMLPs* genes are involved in responding to PaWB phytoplasma invasion. We confirmed that the transcription of *PfMLP25* is regulated by the transcription factor PfMYB100, and detected interactions between PfMLP25 and PfCDPKa/PfRODa, two key proteins mediating immune system activation during PaWB phytoplasma infection. Collectively, our findings demonstrate that *PfMLP25* enhances pathogen resistance and promotes immune system activation in Paulownia. This study provides a novel insight into the biological functions and potential molecular mechanisms of PfMLP25 in modulating plant responses to pathogen invasion.

## SUPPLEMENTARY DATA

Supplementary data to this article can be found online.

## Data Availability

All data generated in this study were obtained from the National Center for Biotechnology Information database (www.ncbi.nlm.nih.gov, accession numbers: SRR11787882–SRR11787971).
