# Supplementary material for: Comprehensive analysis of the MLP genes in Paulownia fortunei and functional characterization of PfMLP25 in response to pathogen invasion
Source: For Res (Fayettev). 2026 Mar 31;6:e009. doi: 10.48130/forres-0026-0008 (PMC13191360; doi:10.48130/forres-0026-0008)

Figure S1. Analyses of chromosomal location, gene structure, and motif between the *PfMLPs* members in *P. fortunei*. A. Distribution of *PfMLP* genes in chromosomes. B. Exon/intron structures of 49 *PfMLPs*. C. Architecture of motifs of 49 *PfMLPs*.

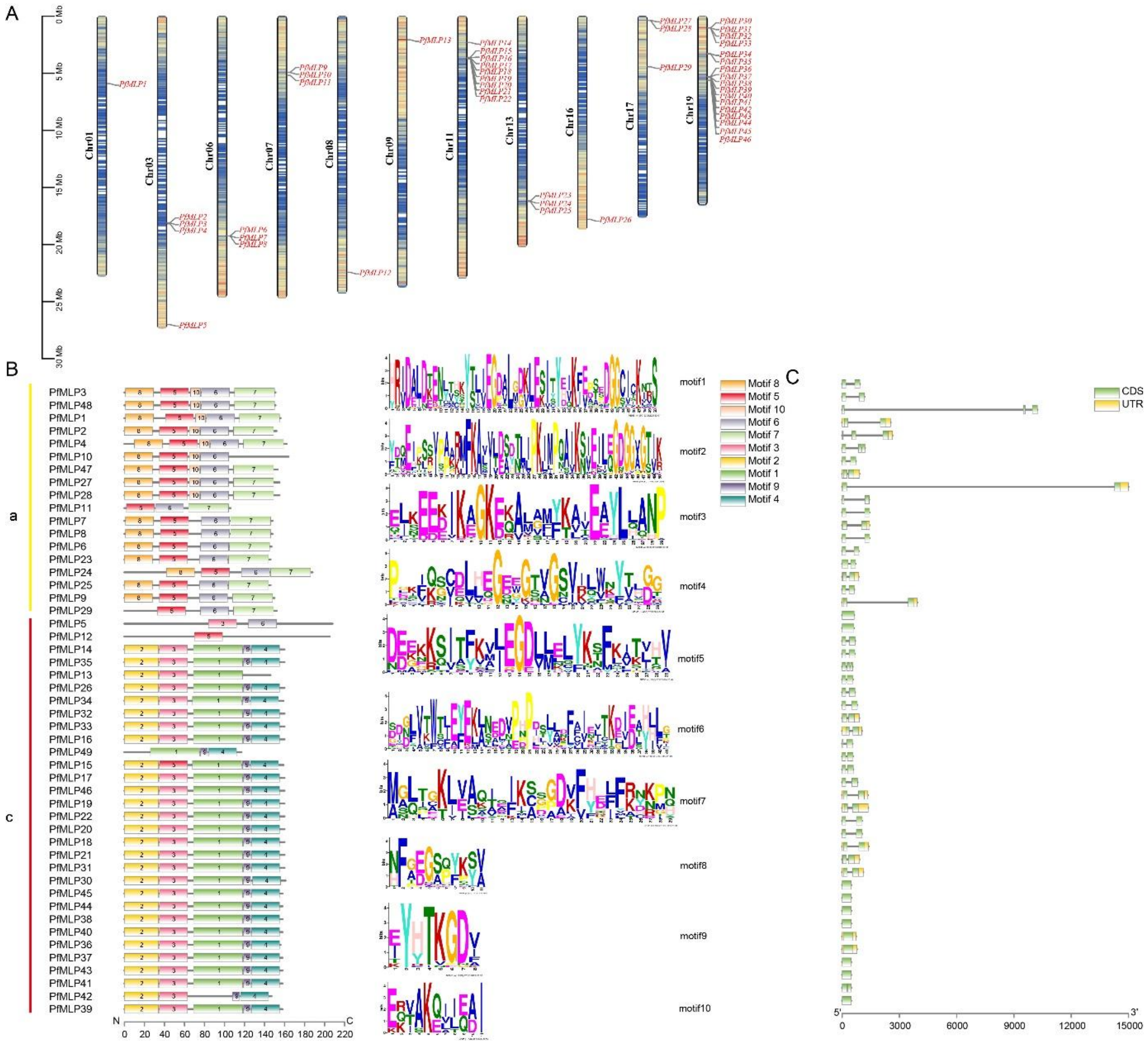

Supplement: Supplementary file 1 — Supplementary data to this article can be found online. [file FR-2026-6-008-S1.zip › 10.48130_forres-0026-0008-Suppl-FigureS1.pdf]
