# Supplementary material for: Comprehensive analysis of the MLP genes in Paulownia fortunei and functional characterization of PfMLP25 in response to pathogen invasion
Source: For Res (Fayettev). 2026 Mar 31;6:e009. doi: 10.48130/forres-0026-0008 (PMC13191360; doi:10.48130/forres-0026-0008)

Figure S2. RT-qPCR of *PfMLP* genes expression. A. The expression level of *PfMLP25* under MMS and Rif treatments. B. Expression level of the *PfMLP* genes under SA treatment. Data are mean ± SD; different letters indicate significant difference based on multiple comparisons (Duncan method) after ANOVA.

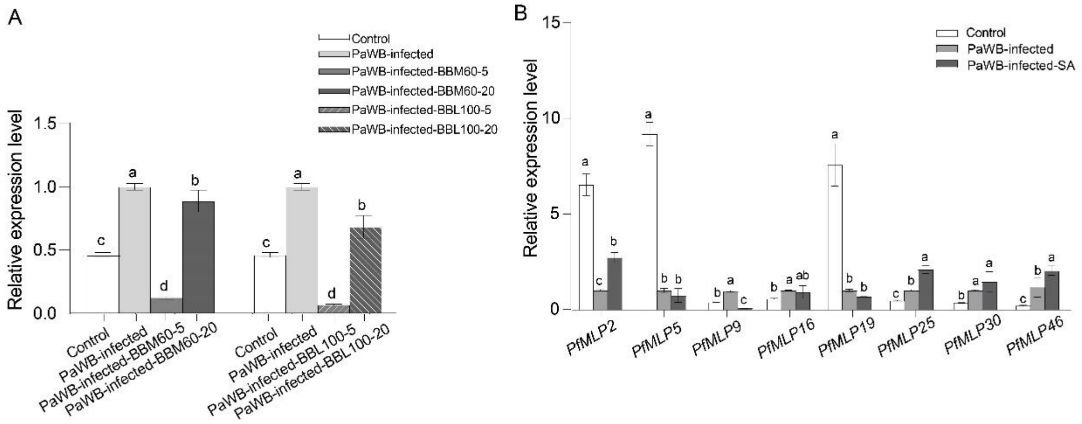

Supplement: Supplementary file 1 — Supplementary data to this article can be found online. [file FR-2026-6-008-S1.zip › 10.48130_forres-0026-0008-Suppl-FigureS2.pdf]
