# Supplementary material for: Comprehensive analysis of the MLP genes in Paulownia fortunei and functional characterization of PfMLP25 in response to pathogen invasion
Source: For Res (Fayettev). 2026 Mar 31;6:e009. doi: 10.48130/forres-0026-0008 (PMC13191360; doi:10.48130/forres-0026-0008)

Figure S3. CREs prediction of 49 *PfMLPs*. The CREs within the 2000 bp promoter region upstream of 49 *PfMLPs* are depicted, with each type of CRE represented by a distinct color.

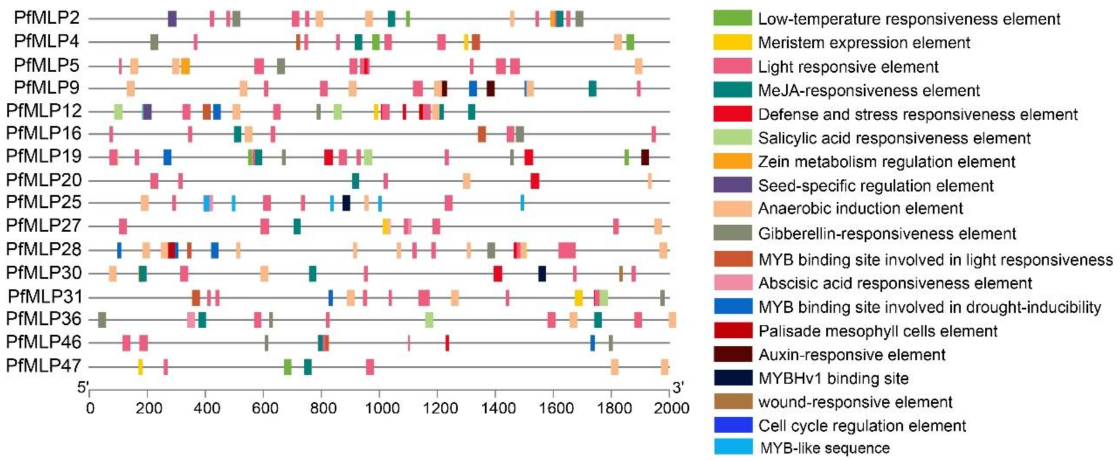

Supplement: Supplementary file 1 — Supplementary data to this article can be found online. [file FR-2026-6-008-S1.zip › 10.48130_forres-0026-0008-Suppl-FigureS3.pdf]
