# Supplementary material for: Comprehensive analysis of the MLP genes in Paulownia fortunei and functional characterization of PfMLP25 in response to pathogen invasion
Source: For Res (Fayettev). 2026 Mar 31;6:e009. doi: 10.48130/forres-0026-0008 (PMC13191360; doi:10.48130/forres-0026-0008)

Figure S4. Subcellular localization of PfMPL25 in *N. benthamiana* leaves. High-resolution laser confocal microscope of leaf tissues transiently expressing PfMLP25-GFP or GFP alone. GFP: green fluorescent protein, RFP: red fluorescence protein, AtSUN1::RFP was utilized as nuclear envelope marker. Bars = 20 µm.

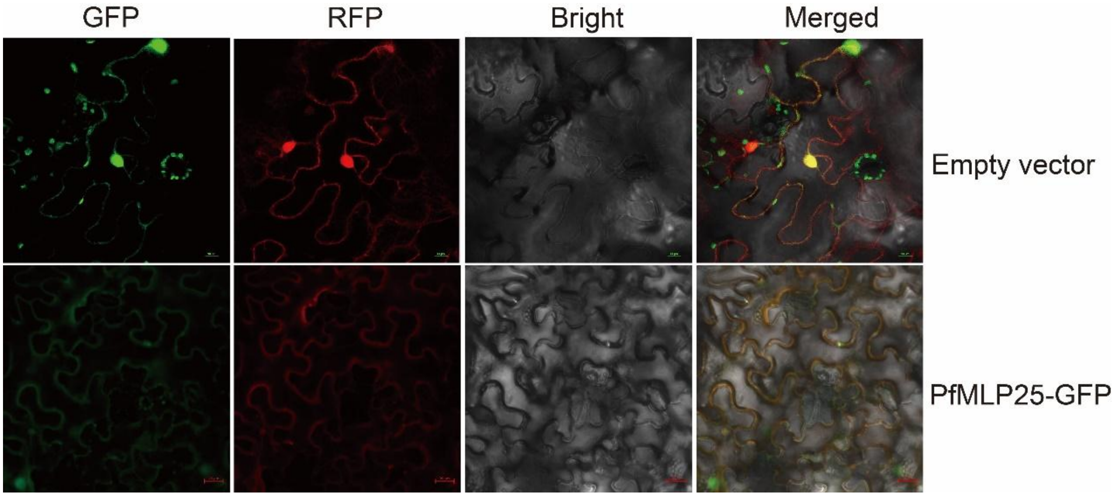

Supplement: Supplementary file 1 — Supplementary data to this article can be found online. [file FR-2026-6-008-S1.zip › 10.48130_forres-0026-0008-Suppl-FigureS4.pdf]
