# Supplementary material for: Comprehensive analysis of the MLP genes in Paulownia fortunei and functional characterization of PfMLP25 in response to pathogen invasion
Source: For Res (Fayettev). 2026 Mar 31;6:e009. doi: 10.48130/forres-0026-0008 (PMC13191360; doi:10.48130/forres-0026-0008)

Figure S5. PfMLP25 pull down MS in paulownia. A. Verification of PfMLP25-pGS21T whole bacteria expression. 0, Whole bacteria not induced;1, Strain 1 induced; 2, Strain 2 induced. B. PfMLP25-GST column purification assay. 0, Fermentation-inducing bacteria ultrasonically broken and centrifuged precipitate; 1, Fermentation-inducing bacteria ultrasonically broken and centrifuged supernatant; 2, Supernatant GST purification column effluent; 3, Elution solution elutes the target protein; 4, Residual protein remaining on the column; M, 116 kDa, 66 kDa, 45 kDa, 35 kDa, 25 kDa, 18 kDa, 14 kDa (from top to bottom). PfMLP25 as bait to perform pull-down analysis diseased paulownia seedlings (C). 0, total tissue protein; 1, target protein pull down assay; 2, control protein pull down assay. M, 116 kDa, 66 kDa, 45 kDa, 35 kDa, 25 kDa, 18 kDa (from top to bottom).

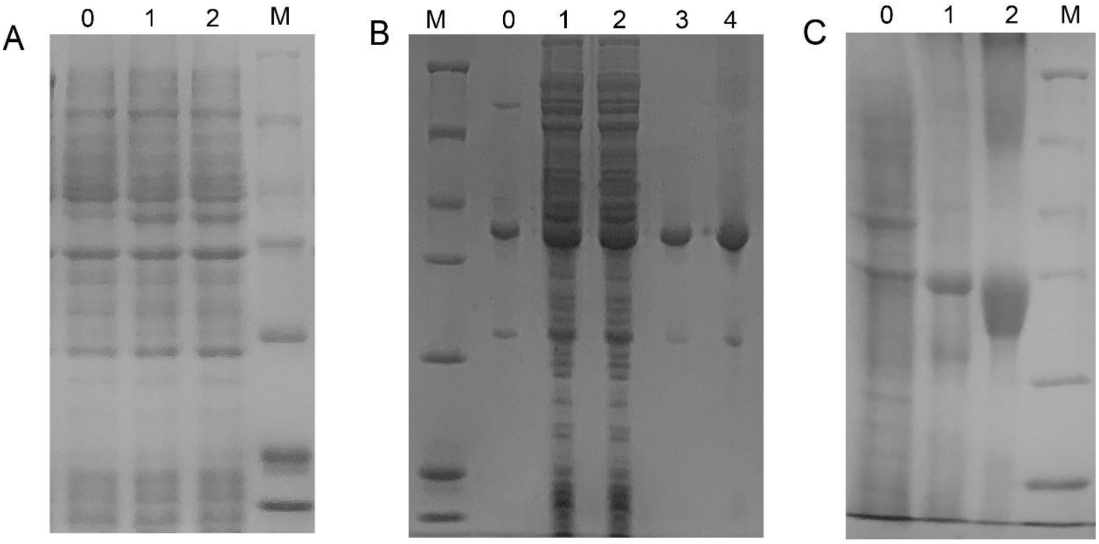

Supplement: Supplementary file 1 — Supplementary data to this article can be found online. [file FR-2026-6-008-S1.zip › 10.48130_forres-0026-0008-Suppl-FigureS5.pdf]
