# Supplementary material for: Comprehensive analysis of the MLP genes in Paulownia fortunei and functional characterization of PfMLP25 in response to pathogen invasion
Source: For Res (Fayettev). 2026 Mar 31;6:e009. doi: 10.48130/forres-0026-0008 (PMC13191360; doi:10.48130/forres-0026-0008)

Figure S6. Functional analysis of proteins interacting with PfMLP25. (A). KEGG pathway enrichment analysis of PfMLP25-interacting proteins. (B). The histogram chart illustrates the distribution of PfMLP25-interacting proteins identified via GST pull-down MS screening, categorized based on function analysis.

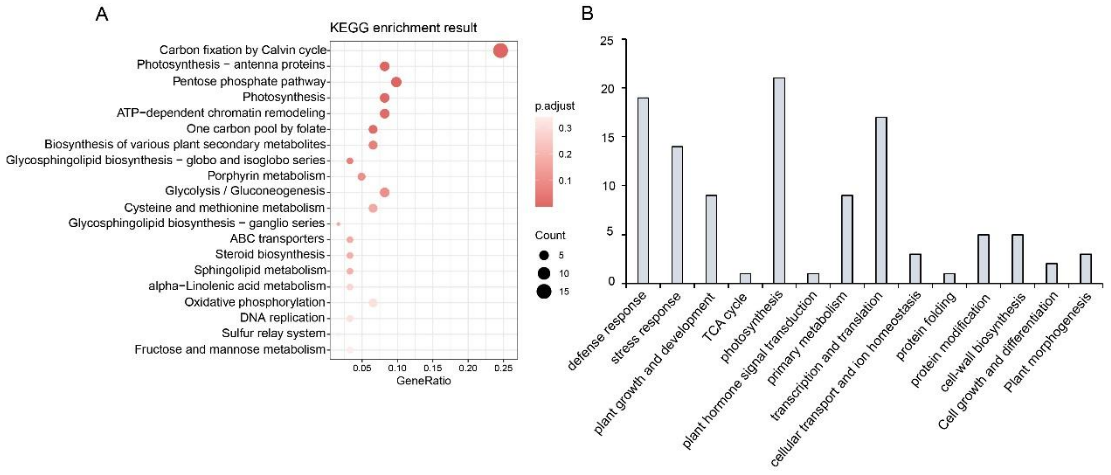

Supplement: Supplementary file 1 — Supplementary data to this article can be found online. [file FR-2026-6-008-S1.zip › 10.48130_forres-0026-0008-Suppl-FigureS6.pdf]
