# Supplementary material for: Comprehensive analysis of the MLP genes in Paulownia fortunei and functional characterization of PfMLP25 in response to pathogen invasion
Source: For Res (Fayettev). 2026 Mar 31;6:e009. doi: 10.48130/forres-0026-0008 (PMC13191360; doi:10.48130/forres-0026-0008)
Supplement: Supplementary file 1 — Supplementary data to this article can be found online. [file FR-2026-6-008-S1.zip › 10.48130_forres-0026-0008-Suppl-TableS1.pdf]

Table S1 Primers used to generate the vectors

Primer name	Primer sequence (5'-3')
pGADT7-PfPUBb-F	GTACCAGATTACGCTCATATGATGTATCTGAGTACAACAAGGCAACC
pGADT7-PfPUBb-R	ACGATTCATCTGCAGCTCGAGCTAGTTTGGAAAGATACCGGAGAAG
pGADT7-PfPUBa-F	GTACCAGATTACGCTCATATGATGGCTGGAAGCTGGGATG
pGADT7-PfPUBa-R	ACGATTCATCTGCAGCTCGAGTCCCATGTTTGGAAAGATACCAG
pGADT7-PfMLP25-F	GTACCAGATTACGCTCATATGATGTCTCAGACAGCTAAGA
pGADT7-PfMLP25-R	ACGATTCATCTGCAGCTCGAGATGGGTAAGAAGGTAAAGATCC
pGADT7-PfRODa-F	GTACCAGATTACGCTCATATGATGAAGAAATCCCTTCTCTTAC
pGADT7-PfRODa-R	ACGATTCATCTGCAGCTCGAGTTTTCTCGCTTTTCTTCCCCG
pGADT7-PfMYB39-F	GTACCAGATTACGCTCATATGATGGGCAGGCCGCCTTGC
pGADT7-PfMYB39-R	ACGATTCATCTGCAGCTCGAGAAATAATTCCGGATTCTCATC
pGADT7-PfMYB41-F	GTACCAGATTACGCTCATATGATGGATAAAAAACCATGCAAATCTC
pGADT7-PfMYB41-R	ACGATTCATCTGCAGCTCGAGTTCTCCATTAAGTAACTGTAGGGACCA
pGADT7-PfMYB57-F	GTACCAGATTACGCTCATATGATGGGAAGACCACCTTGCTGT
pGADT7-PfMYB57-R	ACGATTCATCTGCAGCTCGAGGAACAAATCAGCAGTTTCACC
pGADT7-PfMYB74-F	GTACCAGATTACGCTCATATGATGGAACAACATGCTAAAGTT
pGADT7-PfMYB74-R	ACGATTCATCTGCAGCTCGAGAACATTGCTGTTGAACTGCTG
pGADT7-PfMYB100-F	GTACCAGATTACGCTCATATGATGGGCAGGCCACCTTGC
pGADT7-PfMYB100-R	ACGATTCATCTGCAGCTCGAGAAATAAATTTGGATTTTCATC
pGADT7-PfMYB104-F	GTACCAGATTACGCTCATATGATGGGAAGGCCACCTTGCTGT
pGADT7-PfMYB104-R	ACGATTCATCTGCAGCTCGAGGAACAAATCAGCAGTTTCCCCC
pGADT7-PfMYB128-F	GTACCAGATTACGCTCATATGATGGATCAAGATGCTAAAGTT
pGADT7-PfMYB128-R	ACGATTCATCTGCAGCTCGAGAGCATTGCTGTTGAATTGCTG
pGADT7-PfMLP25(1-45)-F	GTACCAGATTACGCTCATATGATGTCTCAGACAGCTAAGA
pGADT7-PfMLP25(1-45)-R	ACGATTCATCTGCAGCTCGAGAAGTAATTTAACATTTTTG
pGADT7-PfMLP25(46-120)-F	GTACCAGATTACGCTCATATGATGGAAGGAGAGGAAGGT
pGADT7-PfMLP25(46-120)-R	ACGATTCATCTGCAGCTCGAGATCATTCGCTTTCTCAAACTC
pGADT7-PfMLP25(121-146)-F	GTACCAGATTACGCTCATATGATGTGTGTGCCAAACCCAGAC
pGADT7-PfMLP25(121-146)-R	ACGATTCATCTGCAGCTCGAGATGGGTAAGAAGGTAAAGATC
pGADT7-PfMLP25(1-120)-F	GTACCAGATTACGCTCATATGATGTCTCAGACAGCTAAGA
pGADT7-PfMLP25(1-120)-R	ACGATTCATCTGCAGCTCGAGATCATTCGCTTTCTCAAACTC
pSAK277-PfMYB39-FLAG-F	TCCAAAGAATTCCCCGGTACCATGGGCAGGCCGCCTTGC
pSAK277-PfMYB39-FLAG-R	ATGATCTTTGTAATCCTCGAGAAATAATTCCGGATTCTCATC
pSAK277-PfMYB41-FLAG-F	TCCAAAGAATTCCCCGGTACCATGGATAAAAAACCATGCAAATCTC
pSAK277-PfMYB41-FLAG-R	ATGATCTTTGTAATCCTCGAGTTCTCCATTAAGTAACTGTAGGGACCA
pSAK277-PfMYB57-FLAG-F	TCCAAAGAATTCCCCGGTACCATGGGAAGACCACCTTGCTGT
pSAK277-PfMYB57-FLAG-R	ATGATCTTTGTAATCCTCGAGGAACAAATCAGCAGTTTCACC
pSAK277-PfMYB74-FLAG-F	TCCAAAGAATTCCCCGGTACCATGGAACAACATGCTAAAGTT
pSAK277-PfMYB74-FLAG-R	ATGATCTTTGTAATCCTCGAGAACATTGCTGTTGAACTGCTG
pSAK277-PfMYB100-FLAG-F	TCCAAAGAATTCCCCGGTACCATGGGCAGGCCACCTTGC
pSAK277-PfMYB100-FLAG-R	ATGATCTTTGTAATCCTCGAGAAATAAATTTGGATTTTCATC
pSAK277-PfMYB104-FLAG-F	TCCAAAGAATTCCCCGGTACCATGGGAAGGCCACCTTGCTGT
pSAK277-PfMYB104-FLAG-R	ATGATCTTTGTAATCCTCGAGGAACAAATCAGCAGTTTCCCCC
pSAK277-PfMYB128-FLAG-F	TCCAAAGAATTCCCCGGTACCATGGATCAAGATGCTAAAGTT

pSAK277-PfMYB128-FLAG-R	ATGATCTTTGTAATCCTCGAGAGCATTGCTGTTGAATTGCTG
pSAK277-PfMLP25-GFP-F	GTGGATCCAAAGAATTCATGTCTCAGACAGCTAAGATTGAGGC
pSAK277-PfMLP25-GFP-R	CTCCTTTACCCATGAATTCATGGGTAAGAAGGTAAAGATCCAG
pGBKT7-PfMLP25-F	GAGGAGGACCTGCATATGATGTCTCAGACAGCTAAGA
pGBKT7-PfMLP25-R	ACGGATCCCCGGGAATTCATGGGTAAGAAGGTAAAGATCC
pGBKT7-PfRODa-F	TCAGAGGAGGACCTGCATATGATGAAGAAATCCCTTCTCTTAC
pGBKT7-PfRODa-R	TCGACGGATCCCCGGGAATTCTTTTCTCGCTTTTCTTCCCCG
pGBKT7-PfCDPKa-F	TCAGAGGAGGACCTGCATATGATGGGACTTTGTCATGGAAAACCC
pGBKT7-PfCDPKa-R	TCGACGGATCCCCGGGAATTCTGCCTTCTGAAATGTGCGTGAGGA
pGBKT7-PfMYB39-F	TCAGAGGAGGACCTGCATATGATGGGCAGGCCGCCTTGC
pGBKT7-PfMYB39-R	TCGACGGATCCCCGGGAATTCAAATAATTCCGGATTCTCATC
pGBKT7-PfMYB41-F	TCAGAGGAGGACCTGCATATGATGGATAAAAAACCATGCAAATCTC
pGBKT7-PfMYB41-R	TCGACGGATCCCCGGGAATTCTTCTCCATTAAGTAACTGTAGGGACCA
pGBKT7-PfMYB100-F	TCAGAGGAGGACCTGCATATGATGGGCAGGCCACCTTGC
pGBKT7-PfMYB100-R	TCGACGGATCCCCGGGAATTCAAATAAATTTGGATTTTCATC
PHIs-PfMLP25Pro-F	CGACTCACTATAGGGCGAATTCATGTATTTGTAATTTTATTTCAAA
PHIs-PfMLP25Pro-R	ATTCGCGAACGCGTGAGCTCGTGCTAAAGACCAGAAATATAAGT
PfMLP25-ECN-F	AGTGGTCTCTGTCCAGTCCTATGTCTCAGACAGCTAAGA
PfMLP25-ECN-R	GGTCTCAGCAGACCACAAGTATGGGTAAGAAGGTAAAGATCC
PfMYB39-ECN-F	AGTGGTCTCTGTCCAGTCCTATGGGCAGGCCGCCTTGC
PfMYB39-ECN-R	GGTCTCAGCAGACCACAAGTAAATAATTCCGGATTCTCATC
PfMYB100-ECN-F	AGTGGTCTCTGTCCAGTCCTATGGGCAGGCCACCTTGC
PfMYB100-ECN-R	GGTCTCAGCAGACCACAAGTAAATAAATTTGGATTTTCATC
PfMYB41-ENN-F	AGTGGTCTCTGTCCAGTCCTATGGATAAAAAACCATGCAAATCTC
PfMYB41-ENN-R	GGTCTCAGCAGACCACAAGTTTCTCCATTAAGTAACTGTAGGGACCA
PfRODa-ENN-F	AGTGGTCTCTGTCCAGTCCTATGAAGAAATCCCTTCTCTTAC
PfRODa-ENN-R	GGTCTCAGCAGACCACAAGTTTTCTCGCTTTTCTTCCCCG
PfCDPKa-ENN-F	AGTGGTCTCTGTCCAGTCCTATGGGACTTTGTCATGGAAAACCC
PfCDPKa-ENN-R	GGTCTCAGCAGACCACAAGTTGCCTTCTGAAATGTGCGTGAGGA
PfRODa-ECN-F	AGTGGTCTCTGTCCAGTCCTATGAAGAAATCCCTTCTCTTAC
PfRODa-ECN-R	GGTCTCAGCAGACCACAAGTTTTCTCGCTTTTCTTCCCCG
PfPUBa-ENN-F	AGTGGTCTCTGTCCAGTCCTATGGCTGGAAGCTGGGATG
PfPUBa-ENN-R	GGTCTCAGCAGACCACAAGTTCCCATGTTTGGAAAGATACCAG
RT-PtActin-F	AACATGGGATTGTTAGCAACTGG
RT-PtActin-R	TCCATCACCAGAATCCAGCACA
RT-PfMYB39-F	CAGGGCTGAGGAGATG
RT-PfMYB39-R	AATGGGTATTCCAGTAGTTT
RT-PfMYB41-F	GCACGAGCCAAATGTC
RT-PfMYB41-R	TTAAGTAACTGTAGGGACCAA
RT-PfMYB57-F	TCGGATGTCTCCCAATC
RT-PfMYB57-R	TTTCACCCAAGGCAAT
RT-PfMYB74-F	CACCGCCACTACTTACC
RT-PfMYB74-R	CACCGCCACTACTTACC
RT-PfMYB100-F	CAGGGCTGAGGAGATG
RT-PfMYB100-R	GATGGGTATTCCAGTAGTTC
RT-PfMYB104-F	CGATAAATCCGACACGA

RT-PfMYB104-R	GTAAGTCCAACTGAAGGGT
RT-PfMYB128-F	CGGGTAGGACTGACAATG
RT-PfMYB128-R	CGGTAGTTCCGCTGGT
RT-PfPUBa-F	GCGGTGTTCATCGTGG
RT-PfPUBa-R	TGGACAGGGCAGCAAG
RT-PtBAK1-F	TGGGAGGAATGGCAGAA
RT-PtBAK1-R	GGAGGGATGTGGGATGA
RT-PtBIK1-F	GGAGTGGCTGGCAGAAGTAAA
RT-PtBIK1-R	CACAATGAAGAAACGCAAGACC
RT-PtNLH10-F	GCCGTGGTTCTGACTGT
RT-PtNLH10-R	AGGTTGTAGCGGAGCAT
RT-PtXLG2-F	CCCCTCACTTGGTCATC
RT-PtXLG2-R	ATAATCTCCCTCGCATA
RT-PtNPR1-F	TGGCTAAAGACAGAAGTG
RT-PtNPR1-R	GCAATATGGGAACAATCA
RT-PtMKK4-F	ACGGCATCGCCTACTTG
RT-PtMKK4-R	GCATACCCATCATACAATCCT
RT-PfMLP25-F	ATGTTCTTCCGCTGATA
RT-PfMLP25-R	AAGGGCTACAAAGGTTA
RT-PfMLP2-F	GTTCAGGCACAAACCAC
RT-PfMLP2-R	TCTCCTTCCAGCATCTT
RT-PfMLP5-F	CAACTACCGCTCCGTCAC
RT-PfMLP5-R	GGGTTCGTCTGGCTAAACT
RT-PfMLP9-F	TGTCATGGGTGTTCCTG
RT-PfMLP9-R	CGTCTTCACTTGCCTTC
RT-PfMLP16-F	GATGGTGTTAGTGCTGGAT
RT-PfMLP16-R	CGTAGTTCTCGGTGTCAAT
RT-PfMLP19-F	ATACACCCAAGAAATCAAAC
RT-PfMLP19-R	ATCAAGGGCATCAATCC
RT-PfMLP30-F	ATCCTTGAGGGAGACGG
RT-PfMLP30-R	TGGTATGTGCTGGTTGTTT
RT-PfMLP46-F	TGATCGAAGGAGCTGTG
RT-PfMLP46-R	GTTGGCAAGGAGGTATT
RT-PfActin-F	AATGGAATCTGCTGGAAT
RT-PfActin-R	ACTGAGGACAATGTTACC
