# Supplementary material for: Comprehensive analysis of the MLP genes in Paulownia fortunei and functional characterization of PfMLP25 in response to pathogen invasion
Source: For Res (Fayettev). 2026 Mar 31;6:e009. doi: 10.48130/forres-0026-0008 (PMC13191360; doi:10.48130/forres-0026-0008)
Supplement: Supplementary file 1 — Supplementary data to this article can be found online. [file FR-2026-6-008-S1.zip › 10.48130_forres-0026-0008-Suppl-TableS2.pdf]

Table S2. Identification of PfMLP25-interacting proteins.

#GeneID	nr_annotation
Paulownia_LG5G000013.1	peroxidase 47 [Sesamum indicum]
Paulownia_LG3G001405.1	hypothetical protein MIMGU_mgv1a006323mg [Erythranthe guttata]
Paulownia_LG9G000247.1	ATP-dependent Clp protease ATP-binding subunit clpX-like, mitochondrial [Sesamum indicum]
Paulownia_LG15G000502.1	dehydrin ERD14-like [Sesamum indicum]
Paulownia_LG9G001103.1	copper chaperone [Plantago major]
Paulownia_WTDBG01685G000003.1	protein transport protein SEC31 homolog B [Sesamum indicum]
Paulownia_LG0G000726.1	protein transport protein SEC31 homolog B [Sesamum indicum]
Paulownia_CONTIG01576G000018.1	33 kDa ribonucleoprotein, chloroplastic [Sesamum indicum]
Paulownia_LG18G000338.1	probable histone H2A variant 3 [Sesamum indicum]
Paulownia_CONTIG01580G000085.1	histone H2A-like [Sesamum indicum]
Paulownia_LG11G000432.1	hypothetical protein MIMGU_mgv1a015106mg [Erythranthe guttata]
Paulownia_LG2G000418.1	apolipoprotein D-like [Sesamum indicum]
Paulownia_LG0G001228.1	stromal 70 kDa heat shock-related protein, chloroplastic-like [Sesamum indicum]
Paulownia_LG3G000489.1	stromal 70 kDa heat shock-related protein, chloroplastic [Sesamum indicum]
Paulownia_LG6G000141.1	haloacid dehalogenase-like hydrolase domain-containing protein At3g48420 [Sesamum indicum]
Paulownia_CONTIG01580G000084.1	Histone H2B [Medicago truncatula]
novel_model_1898_5afbe647	histone H2B-like [Sesamum indicum]
Paulownia_TIG00001202G000005.1	Histone H2B [Medicago truncatula]
Paulownia_CONTIG01606G000004.1	histone H2B-like [Sesamum indicum]
Paulownia_LG4G001348.1	probable histone H2B.1 [Nicotiana sylvestris]
Paulownia_LG19G000201.1	histone H2B-like [Sesamum indicum]
Paulownia_LG12G000642.1	Histone H2B [Medicago truncatula]
Paulownia_CONTIG01606G000003.1	histone H2B-like [Sesamum indicum]
Paulownia_WTDBG01886G000002.1	Recombinase A protein (RecA) [Blastocystis hominis]
Paulownia_LG8G001873.1	centromere/kinetochore protein zw10 homolog isoform X2 [Sesamum indicum]
Paulownia_LG6G000049.1	hypothetical protein MIMGU_mgv1a007981mg [Erythranthe guttata]
Paulownia_LG0G001914.1	LOW QUALITY PROTEIN: WD-40 repeat-containing protein MSI3 [Sesamum indicum]
Paulownia_WTDBG00024_ERROPOS5331222G000084.1	S-adenosylmethionine synthase 3 [Sesamum indicum]
Paulownia_LG10G001033.1	hypothetical protein MIMGU_mgv1a014564mg [Erythranthe guttata]
Paulownia_WTDBG00068_ERROPOS550008+G000042.1	putative late blight resistance protein homolog R1A-10 [Sesamum indicum]
novel_model_22_5afbe647	uncharacterized protein LOC105174256 [Sesamum indicum]
Paulownia_LG7G001052.1.2.5afc1dc5	uncharacterized protein LOC105156622 [Sesamum indicum]
Paulownia_LG11G000711.1	uncharacterized protein DDB_G0290685 [Sesamum indicum]
Paulownia_LG5G001638.1	glyceraldehyde-3-phosphate dehydrogenase, cytosolic-like [Sesamum indicum]
novel_model_1692_5afbe647	hypothetical protein MIMGU_mgv1a007931mg [Erythranthe guttata]
Paulownia_LG5G000818.1	hypothetical protein MIMGU_mgv1a012403mg [Erythranthe guttata]
Paulownia_LG10G001232.1	hypothetical protein MIMGU_mgv1a011534mg [Erythranthe guttata]
Paulownia_LG15G001224.1	glyceraldehyde-3-phosphate dehydrogenase B, chloroplastic [Prunus mume]
Paulownia_TIG00007408G000001.1	Atp1, partial (mitochondrion) [Calceolaria integrifolia]
Paulownia_WTDBG01062G000002.1	glyceraldehyde-3-phosphate dehydrogenase A, chloroplastic [Sesamum indicum]
Paulownia_LG10G000782.1	glyceraldehyde-3-phosphate dehydrogenase B, chloroplastic [Prunus mume]
Paulownia_WTDBG01062G000004.1	glyceraldehyde-3-phosphate dehydrogenase A, chloroplastic [Sesamum indicum]
novel_model_1873_5afbe647	glyceraldehyde-3-phosphate dehydrogenase A, chloroplastic [Sesamum indicum]
Paulownia_LG11G000715.1	probable xyloglucan endotransglucosylase/hydrolase protein 7 [Sesamum indicum]
Paulownia_LG14G000725.1	glyceraldehyde-3-phosphate dehydrogenase, cytosolic [Sesamum indicum]
novel_model_605_5afbe647	glyceraldehyde-3-phosphate dehydrogenase A, chloroplastic [Sesamum indicum]
Paulownia_LG6G000755.1	glyceraldehyde-3-phosphate dehydrogenase C [Scoparia dulcis]
Paulownia_LG7G000077.1	glyceraldehyde-3-phosphate dehydrogenase, cytosolic [Sesamum indicum]
Paulownia_LG11G001138.1	glyceraldehyde-3-phosphate dehydrogenase, cytosolic-like [Sesamum indicum]
Paulownia_LG8G000949.1	porphobilinogen deaminase, chloroplastic [Sesamum indicum]
Paulownia_LG16G000420.1	chlorophyll a-b binding protein 8, chloroplastic [Sesamum indicum]
Paulownia_WTDBG01049G000002.1	hypothetical protein MIMGU_mgv1a011534mg [Erythranthe guttata]
Paulownia_LG10G000848.1	ATP synthase delta chain, chloroplastic-like [Sesamum indicum]
Paulownia_LG10G000229.1	photosystem I reaction center subunit II, chloroplastic-like [Sesamum indicum]
Paulownia_LG12G001171.1	ruBisCO large subunit-binding protein subunit beta, chloroplastic [Sesamum indicum]
novel_model_80_5afbe647	hypothetical protein MIMGU_mgv1a007931mg [Erythranthe guttata]
Paulownia_LG16G000816.1	porphobilinogen deaminase, chloroplastic [Sesamum indicum]
Paulownia_LG17G000137.1	major allergen Pru av 1-like [Sesamum indicum]
Paulownia_LG14G000087.1	fruit protein pKIWI502-like [Sesamum indicum]
Paulownia_LG14G000035.1	kirola-like [Sesamum indicum]
Paulownia_LG17G000646.1	major allergen Pru ar 1-like [Sesamum indicum]
Paulownia_LG17G000653.1	major allergen Pru ar 1-like [Sesamum indicum]

Paulownia_LG17G000645.1	major allergen Pru ar 1-like [Sesamum indicum]
Paulownia_LG15G000294.1	hypothetical protein MIMGU_mgv1a006830mg [Erythranthe guttata]
Paulownia_LG6G000950.1	hypothetical protein MIMGU_mgv1a011200mg [Erythranthe guttata]
Paulownia_LG11G000586.1	hypothetical protein MIMGU_mgv1a012403mg [Erythranthe guttata]
novel_model_358_5afbe647	hypothetical protein MIMGU_mgv1a011534mg [Erythranthe guttata]
novel_model_537_5afbe647	DNA replication licensing factor MCM6 [Sesamum indicum]
Paulownia_TIG00000033G000013.1	Quinone oxidoreductase-like protein [Morus notabilis]
Paulownia_TIG00000033G000014.1	Quinone oxidoreductase-like protein [Morus notabilis]
Paulownia_LG19G000273.1	copper chaperone [Jatropha curcas]
Paulownia_LG11G000717.1	lysosomal Pro-X carboxypeptidase [Sesamum indicum]
novel_model_701_5afbe647	hypothetical protein MIMGU_mgv1a015417mg [Erythranthe guttata]
novel_model_1122_5afbe647	hypothetical protein MIMGU_mgv1a015417mg [Erythranthe guttata]
Paulownia_LG14G000747.1	40S ribosomal protein SA-like [Sesamum indicum]
Paulownia_LG5G000624.1	photosystem I reaction center subunit N, chloroplastic [Sesamum indicum]
Paulownia_LG12G000187.1	probable histone H2A variant 3 [Sesamum indicum]
Paulownia_LG7G001468.1	probable histone H2A variant 3 [Sesamum indicum]
Paulownia_LG15G000051.1	CDPK-related kinase 1-like [Sesamum indicum]
Paulownia_LG4G000029.1	S-adenosylmethionine synthase 2 [Nelumbo nucifera]
Paulownia_LG3G000579.1	S-adenosylmethionine synthase 1-like [Sesamum indicum]
Paulownia_LG17G000024.1	S-adenosylmethionine synthase 2 [Sesamum indicum]
Paulownia_LG10G000015.1	S-adenosylmethionine synthase 2 [Sesamum indicum]
Paulownia_LG3G000618.1	S-adenosylmethionine synthase 1-like [Sesamum indicum]
novel_model_665_5afbe647	CDPK-related kinase 1-like [Sesamum indicum]
novel_model_1797_5afbe647	33 kDa ribonucleoprotein, chloroplastic [Sesamum indicum]
Paulownia_CONTIG01578G000026.1	33 kDa ribonucleoprotein, chloroplastic [Sesamum indicum]
Paulownia_LG16G000260.1	hypothetical protein MIMGU_mgv1a003720mg [Erythranthe guttata]
Paulownia_LG4G001436.1	unnamed protein product [Coffea canephora]
Paulownia_TIG00002321G000016.1	hypothetical protein MIMGU_mgv1a010546mg [Erythranthe guttata]
Paulownia_LG14G000881.1	hypothetical protein MIMGU_mgv1a006041mg [Erythranthe guttata]
Paulownia_TIG00017490G000139.1	28 kDa ribonucleoprotein, chloroplastic-like [Sesamum indicum]
Paulownia_LG5G001603.1	28 kDa ribonucleoprotein, chloroplastic-like [Sesamum indicum]
Paulownia_LG19G000168.1	amidase 1 isoform X1 [Sesamum indicum]
Paulownia_LG14G000403.1	intracellular ribonuclease LX-like [Sesamum indicum]
Paulownia_LG0G001934.1	uncharacterized protein LOC105169808 [Sesamum indicum]
Paulownia_LG10G001360.1	psbP domain-containing protein 4, chloroplastic [Sesamum indicum]
Paulownia_LG8G000003.1	coproporphyrinogen-III oxidase 1, chloroplastic [Sesamum indicum]
Paulownia_LG15G000451.1	uncharacterized protein LOC105179348 isoform X3 [Sesamum indicum]
Paulownia_LG8G001301.1	chlorophyll a-b binding protein 21, chloroplastic-like [Sesamum indicum]
Paulownia_LG8G001974.1	chlorophyll a-b binding protein 5, chloroplastic [Sesamum indicum]
Paulownia_LG8G000298.1	chlorophyll a-b binding protein 21, chloroplastic [Sesamum indicum]
Paulownia_LG16G001287.1	chlorophyll a-b binding protein of LHCII type 1-like [Musa acuminata subsp. malaccensis]
Paulownia_LG4G000321.1	RAN GTPase-activating protein 1 [Sesamum indicum]
Paulownia_LG8G000155.1	reticuline oxidase-like protein [Sesamum indicum]
Paulownia_LG18G000913.1	primary amine oxidase-like [Sesamum indicum]
novel_model_622_5afbe647	hypothetical protein MIMGU_mgv1a015105mg [Erythranthe guttata]
Paulownia_LG17G000186.1	hypothetical protein MIMGU_mgv1a015105mg [Erythranthe guttata]
Paulownia_LG13G000696.1	hypothetical protein MIMGU_mgv1a015105mg [Erythranthe guttata]
Paulownia_WTDBG01390G000004.1	hypothetical protein MIMGU_mgv1a006687mg [Erythranthe guttata]
Paulownia_WTDBG01390G000003.1	hypothetical protein MIMGU_mgv1a006687mg [Erythranthe guttata]
Paulownia_LG8G000050.1	protein CHROMATIN REMODELING 4 [Sesamum indicum]
Paulownia_LG15G001218.1	U-box domain-containing protein 44 [Sesamum indicum]
novel_model_293_5afbe647	protein NLP9 [Sesamum indicum]
Paulownia_LG10G000789.1	U-box domain-containing protein 44 [Sesamum indicum]
Paulownia_LG10G000616.1	protein NLP9 [Sesamum indicum]
Paulownia_LG7G000666.1	cell division cycle and apoptosis regulator protein 1 isoform X2 [Sesamum indicum]
Paulownia_LG3G000906.1	sphingoid long-chain bases kinase 1-like [Sesamum indicum]
Paulownia_LG0G001260.1	protein EI24 homolog isoform X1 [Sesamum indicum]
Paulownia_LG0G000795.1	sphingoid long-chain bases kinase 1-like [Sesamum indicum]
Paulownia_LG0G000737.1	cytokinin dehydrogenase 6-like [Sesamum indicum]
Paulownia_LG14G000388.1	uncharacterized protein LOC105160078, partial [Sesamum indicum]
Paulownia_LG5G001321.1	hypothetical protein MIMGU_mgv1a017954mg, partial [Erythranthe guttata]
novel_model_1347_5afbe647	pentatricopeptide repeat-containing protein At1g62680, mitochondrial-like [Sesamum indicum]
Paulownia_CONTIG01580G000301.1	pentatricopeptide repeat-containing protein At1g62680, mitochondrial-like [Sesamum indicum]
Paulownia_LG14G000495.1	unnamed protein product [Coffea canephora]
Paulownia_TIG00017490G000030.1	hypothetical protein MIMGU_mgv1a024403mg, partial [Erythranthe guttata]
Paulownia_LG5G001445.1	ABC transporter B family member 15-like [Solanum tuberosum]
Paulownia_LG6G001424.1	hypothetical protein MIMGU_mgv1a015798mg [Erythranthe guttata]

Paulownia_LG7G001055.1	geraniol 8-hydroxylase-like [Sesamum indicum]
Paulownia_LG10G001055.1	hypothetical protein MIMGU_mgv1a005614mg [Erythranthe guttata]
Paulownia_LG17G000513.1	conserved oligomeric Golgi complex subunit 1 [Sesamum indicum]
Paulownia_CONTIG01576G000009.1	probable receptor protein kinase TMK1 [Sesamum indicum]
Paulownia_LG4G000533.1	myosin-6-like [Sesamum indicum]
Paulownia_LG3G001328.1	LOW QUALITY PROTEIN: uncharacterized protein LOC105172331 [Sesamum indicum]
Paulownia_LG7G000515.1	ATP synthase subunit beta, mitochondrial-like [Sesamum indicum]
Paulownia_LG14G000088.1	ATP synthase subunit beta, mitochondrial [Sesamum indicum]
Paulownia_WTDBG01736G000003.1	atpB gene product (chloroplast) [Schizomeris leibleinii]
Paulownia_LG9G000640.1	dirigent protein 22-like [Sesamum indicum]
Paulownia_LG0G001957.1.1.5afc1db1	uncharacterized protein LOC105169823 isoform X3 [Sesamum indicum]
novel_model_700_5afbe647	hypothetical protein MIMGU_mgv1a015413mg [Erythranthe guttata]
Paulownia_LG3G000035.1	hypothetical protein L484_014336 [Morus notabilis]
Paulownia_LG18G000261.1	hypothetical protein MIMGU_mgv1a007287mg [Erythranthe guttata]
Paulownia_LG6G000521.1	Solanum lycopersicum RNA polymerase-associated protein LEO1 (LOC101254888), transcript variant X2,
Paulownia_LG16G000769.1	hypothetical protein MIMGU_mgv1a015209mg [Erythranthe guttata]
Paulownia_LG5G000874.1	DNA ligase 1 isoform X1 [Sesamum indicum]
Paulownia_LG8G000848.1	nuclear inhibitor of protein phosphatase 1 [Sesamum indicum]
Paulownia_LG0G001354.1	beta-xylosidase/alpha-L-arabinofuranosidase 2-like [Sesamum indicum]
novel_model_1879_5afbe647	uncharacterized protein LOC105170210 [Sesamum indicum]
Paulownia_LG14G000774.1	uncharacterized protein LOC105168422 [Sesamum indicum]
Paulownia_LG4G001647.1	uncharacterized protein LOC105170210 [Sesamum indicum]
Paulownia_LG10G001292.1	cytoplasmic tRNA 2-thiolation protein 1 [Sesamum indicum]
Paulownia_LG8G001932.1	U-box domain-containing protein 4 [Sesamum indicum]
Paulownia_LG16G000353.1	G-type lectin S-receptor-like serine/threonine-protein kinase SD2-5 [Sesamum indicum]
Paulownia_LG16G000352.1	G-type lectin S-receptor-like serine/threonine-protein kinase SD2-5 [Sesamum indicum]
Paulownia_LG10G000258.1	pentatricopeptide repeat-containing protein At3g22670, mitochondrial [Sesamum indicum]
